# Teledentistry for Improving Access To, and Quality of Oral Health Care: Overview of Systematic Reviews and Meta-Analyses

**DOI:** 10.2196/65211

**Published:** 2025-07-30

**Authors:** Pascaline Kengne Talla, Paul Allison, André Bussières, Anisha Rodrigues, Frédéric Bergeron, Nicolas Giraudeau, Elham Emami

**Affiliations:** 1 Faculty of Dental Medicine and Oral Health Sciences, McGill University, 2001 McGill College Ave, Montreal, QC, H3A 1G1, Canada, 1 (514) 398-7203; 2 Faculty of Medicine and Health Sciences, School of Physical and Occupational Therapy, McGill University, Montreal, QC, Canada; 3 Département Chiropratique, Université du Québec à Trois-Rivières, Trois-Rivières, QC, Canada; 4 Department of Preventive and Social Medicine, Université Laval, Quebec City, QC, Canada; 5 CEPEL, CNRS, University of Montpellier, Montpellier, France

**Keywords:** teledentistry, dental health, mobile health, digital health, oral health, quality of care, access to care, digital interventions, overview, systematic review, meta-analysis, PRISMA, Preferred Reporting Items for Systematic reviews and Meta-Analyses

## Abstract

**Background:**

Digital interventions including teledentistry are promising approaches to address some of the inadequacies of health care systems. Despite existing systematic reviews (SRs) on the benefits, implementation challenges, accuracy, and effectiveness of teledentistry, a comprehensive synthesis of evidence on its impacts requires further analysis.

**Objective:**

The purpose of this overview of SRs is to summarize evidence on the impacts of teledentistry in promoting access to and enhancing the quality of oral health care.

**Methods:**

We searched electronic databases in MEDLINE (Ovid), Embase (Embase.com), CINAHL (EBSCO), Web of Science, Cochrane Library, and Epistemonikos from inception to March 2024, without date and language restrictions, to identify SRs and meta-analyses. Two independent reviewers performed data selection following the PICOSS (population, intervention, comparison, outcome, and study design) format, as well as the data extraction. We conducted quality assessments using both (A MeaSurement Tool to Assess Systematic Reviews-2) AMSTAR 2 and ROBIS (Risk Of Bias In Systematic reviews) tools. The certainty of evidence and the overlap of the primary studies included in the SRs were assessed. Results were presented in tables and graphs. A narrative synthesis was performed.

**Results:**

The search yielded 1020 articles, of which 30 SRs were included in the overview. The number of participants across these reviews ranged from 130 to 7913 people. All dimensions of the quality of care were addressed to varying extents, with the domains of effectiveness (22/30, 73%), patient-centered care (14/30, 47%), and efficiency (11/30, 37%) being the most extensively studied. Teledentistry addressed public health challenges by improving access to oral health care and reducing inequities (9/30, 30%) for vulnerable people. The major teledentistry applications were teleconsultation (13/30, 43%), and telediagnosis (9/30, 33%). Teledentistry enhanced patient-clinician communication, quality of life, and care experiences for both patients and providers. However, multilevel barriers must be addressed to ensure its successful implementation (7/30, 23%). Meanwhile, patient safety (8/30, 27%) and equity (1/30, 10%) were the least explored domains, with few reviews addressing adverse outcomes, as well as concerns related to data privacy (3/30, 10%) and confidentiality (2/30, 6%). Several SRs exhibited a critically low to low methodological quality (25/30, 83%) and a high risk of bias (8/30, 27%). The overlap (corrected covered area) of the primary studies in all the SRs was slight (30/30, 2.3%), while it was moderate (11/30, 5.7%) for SRs with meta-analyses.

**Conclusions:**

The findings of this overview suggest that teledentistry is an effective and efficient alternative to in-person oral health care. However, significant concerns regarding the quality of the reviews highlight an urgent need for more methodologically rigorous studies to generate robust and reliable evidence. This is particularly essential to better understand teledentistry’s potential to enhance overall health outcomes and ensure equitable access to care, thereby providing a stronger foundation to guide clinical practices and inform policy decisions.

## Introduction

### Background

Oral diseases globally affect more than 3.5 billion people worldwide, highlighting the need for interventions to improve accessibility to and affordability of oral health care [1]. Information and communication technologies (ICTs) offer promising approaches to address these issues, promote high-value care [2-4], and improve the quality of health care [5,6]. Their importance became particularly evident during the COVID-19 pandemic [7], which accelerated their adoption and underscored their critical role in enhancing the delivery of health care services. Teledentistry, a specific application of ICTs in health care, facilitates remote oral health care delivery by enabling communication and collaboration among oral health care providers (OHCPs), and other health care professionals, their patients or caregivers, ultimately improving patient outcomes [8,9]. It enables interactions between patients and other members of the circle of care in dentistry, facilitates screening and diagnosis of oral diseases, and enhances patients’ monitoring, treatment planning, and management of oral health care [10-12]. Emerging evidence suggests that teledentistry has positive impacts for patients, OHCPs, health care providers, and at the societal level [13-17]. In addition, several studies have reported the implementation challenges of teledentistry [8,13,18-23]; these include, for instance, the lack of policy and guidelines, insufficient training, and limited knowledge and digital literacy.

Three previous systematic reviews of systematic reviews (SRs) on teledentistry have a limited scope [24-26]. One overview focused on specific outcomes related to screening, diagnosis, and clinical outcomes, and the domain of teledentistry effectiveness, the second emphasized teleorthodontics, and the third on the effects of teledentistry on costs. All these overviews only involved a small number of SRs, and all 3 have only assessed the methodological quality of the included reviews. These limitations underscore the need for further evidence on health, access to care, and health-related behaviors from patients, families, and OHCPs’ perspectives. Consequently, there is a critical need for a comprehensive synthesis of existing SRs on teledentistry using a rigorous methodology to compile and contrast the evidence, evaluate their quality and the risk of bias, and assess the level of evidence supporting teledentistry using valid measures [27-29]. Given these observations and the growing body of knowledge on teledentistry [7], the purpose of this overview was to summarize evidence from SRs examining the impact of teledentistry on access to and the quality of care. Accurate information from this overview will inform clinical practice and policy decision-making and could assist in the development of guidelines. They may support OHCPs with the teledentistry implementation. In addition, this overview will help to identify gaps in informing future research needs, opportunities, and directions on teledentistry.

### Research Question

We will answer the following research question: “From the perspective of a range of stakeholders, to what extent is teledentistry effective in improving access to, and quality of oral health care, while reducing related costs?”

## Methods

### Study Design

Throughout the overview, the Preferred Reporting Items for Systematic Reviews and Meta-Analyses (PRISMA) guidance was followed. The completed PRISMA checklist (Checklist 1) is included with the paper.

### Search Strategy

The search strategy was developed by the research team with the collaboration of an expert librarian. The bibliographic search was carried out without any restrictions (language, age of participants, etc). A variety of search terms and concepts, including “Teledentistry,” “Dental Health Services,” “Telemedicine,” “Telehealth,” “Remote care,” “Mobile phone,” “mHealth,” “e-health,” “Systematic review,” and “Meta-analysis” were used along with Boolean operators. Five electronic databases, namely, MEDLINE (Ovid), Embase, CINAHL (EBSCO), Web of Science, Cochrane Library/Wiley, and Epistemonikos were searched from inception until March 2024. In addition, we checked the references of the included reviews. Full details of the search strategy, including the list of search terms, can be found in Multimedia Appendix 1. The search strategy followed the “PICOSS” (Participants, Intervention, Comparator, Outcome, Study design, and Setting) format. Participants included patients, informal caregivers, and OHCPs. Interventions were synchronous or asynchronous modalities of teledentistry [16]. Comparators were usual care or no intervention. The outcomes of interest were related to access and quality of care. Quality is a complex and multidimensional concept with no agreed definition in dentistry, and a scarcity of valid and reliable measures to assess quality [30,31]. According to the National Academy of Medicine, previously the Institute of Medicine (IoM) [32], the quality of care includes safety, effectiveness, timeliness, patient-centeredness, efficiency, and equity [32]. Among these domains of quality, safety refers to avoiding harm (eg, infections, privacy, and adverse effects) to patients resulting from (oral) health care; effectiveness includes providing (oral) health care services based on scientific knowledge and to those likely to benefit (avoiding overuse, underuse, misuse, and accuracy); patient-centeredness is providing (oral) health care that is respectful of and responsive to individual patient preferences, needs, and values (eg, values and communication); timeliness means reducing waiting times and sometimes delays for both those who receive and those who give (oral) health care; efficiency is avoiding waste for (oral) health care delivery (eg, equipment, supplies, and energy); and equity refers to providing (oral) health care that does not vary in quality because of personal characteristics such as gender, ethnicity, geographic location, and socioeconomic status. Additional outcomes, such as the environmental impact, the psychosocial and cultural well-being, will be reported if available.

Any dental care settings, geographical regions, and countries were included. Regarding study designs, we included all SRs with or without meta-analysis (SR-MAs). We excluded SRs that performed their search in a single database only, duplicate publications, conference abstracts and literature reviews, and SRs and SR-MAs of animal or in vitro studies. We also excluded SRs lacking a formal methodological quality or risk of bias assessment, which is critical for evaluating the credibility and quality of evidence generated by the SR [33,34]. In addition, we excluded a SR in which the authors mentioned the assessment of the risk of bias in the methods but did not report the results and also did not respond to 2 follow-up emails [35].

### Study Selection

Two independent reviewers [PKT and AR] screened and selected the included studies using Covidence software (Covidence systematic review software, Veritas Health Innovation) [36]. Disagreements at each stage of study selection were resolved through discussion or consultation with a third reviewer [EE]. The decisions and reasons for exclusion were recorded in Covidence software. Refer to Multimedia Appendix 2 for the list of excluded studies with reasons for exclusion.

### Deviation From the Protocol

We did not have any language restrictions during the review process, as mentioned in the protocol [37]. We used translation software when it was needed but found no relevant SR for inclusion. In addition, we did not perform a new Grading of Recommendations, Assessment, Development, and Evaluations (GRADE) for specific outcomes, due to limited available resources.

### Data Extraction

Two research team members [PKT and AR] independently extracted data using a form in Excel (Microsoft Corporation), following the Joanna Briggs Institute’s data extraction form for review of SRs [38]. Any discrepancies were resolved through discussion or by consultation with a third reviewer [EE]. The extracted information included review characteristics, participants, intervention and comparators, outcomes, and methods. Particularly for SR-MAs, we extracted details including sensitivity or subgroup analysis, certainty of evidence, and tests of heterogeneity. Other details collected included main conclusions, limitations, next steps, funding, and conflicts of interest.

### Quality Assessment of Reviews

Empirical evidence is lacking on the optimal tool for assessing risk of bias or methodological in overviews of reviews [28]. Two independent reviewers performed the quality assessment of included SRs using 2 complementary tools: (1) the AMSTAR-2 checklist (A MeaSurement Tool to Assess Systematic Reviews-2) to evaluate the methodological quality and the flaws in the conduct of the reviews [39]; and (2) the ROBIS (Risk Of Bias In Systematic reviews) tool that focuses mainly on the assessment of the level of bias within SRs [40]. We used both tools to conduct a comprehensive assessment of the quality of SRs, leveraging their strengths to offset each other’s limitations. For example, AMSTAR 2 evaluates aspects such as the list of excluded studies with reasons for exclusion and declarations of conflicts of interest, which could introduce bias. In contrast, the ROBIS tool provides a more detailed evaluation of the synthesis process [41]. Before the assessment, a calibration exercise involving 2 independent reviewers [PKT and AR] was conducted through a pilot assessment of 10% of the included SRs using both tools. We assessed all 16 items of AMSTAR 2 for SR-MAs. However, for SRs without MA, we excluded items 11, 12, and 15, as these items are related to MAs [39]. Any discrepancies between the reviewers during the process were resolved by discussion or consultation with a third reviewer [EE].

### Data Synthesis

We conducted a narrative synthesis of the findings. We compiled a list of the primary studies included in all the SRs with or without meta-analysis. We considered clinical, methodological, and statistical heterogeneity. Heterogeneity or between-study variability describes differences in underlying study parameters such as participants, types of outcome measurements, and intervention characteristics termed clinical heterogeneity; variability in the study designs and its quality called methodological heterogeneity; and the variability in effects between referring to statistical heterogeneity [42]. Methodological and clinical sources of heterogeneity contribute to the magnitude and presence of statistical heterogeneity [42]. We applied the following thresholds for the interpretation of the reported *I*^2^ statistic that assesses heterogeneity [42,43] in any reported meta-analysis: 0%‐40% might not be important; 30%‐60% represents moderate heterogeneity; 50%‐90% represents a substantial heterogeneity; and 75%‐100% represents a considerable heterogeneity. When there were no pooled results available, we reported the mean and SD values or the odds ratios or risk ratios and associated CIs, or data regarding sensitivity and specificity whenever available. We did not conduct any assessment of the certainty of evidence. However, we reported the GRADE assessment, or any evaluation of the strength of the evidence as assessed by the authors of the SRs.

One of the major concerns of overviews is the inclusion of some primary studies contributing more than once to the findings. Even though there is no agreement in the literature on how to manage an overlap, assessing its impact is important. The overlap of the included primary studies in the SRs was assessed by calculating the covered area (CA; percentage overlap) and the corrected covered area (CCA) [44,45]. The CA is the total number of studies divided by the product of the rows in columns of the citation matrix. The CCA represents the area (of the citation matrix) that is covered after eliminating the inclusion of all primary studies the first time they are counted. It is calculated using the formula:


CCA=N−r/r/(r∗c−r)

where N is the total number of included primary studies; r is the number of rows (number of primary studies); and c is the number of columns (total number of SRs). The Graphical Representation of Overlap for Overviews (GROOVE) tool [46] was used to explore and present the nature and the extent of the overlap among the primary studies in included SRs. The thresholds for its interpretation were: (0%‐5%—slight, 6%‐10%—moderate, 11%‐15%—high, >15%—very high) [44,47]. We organized data extracted from the included SRs into diagrams and tables. In addition, we summarized the findings and carried out a narrative synthesis.

## Results

### Data selection and management

**Figure 1.**
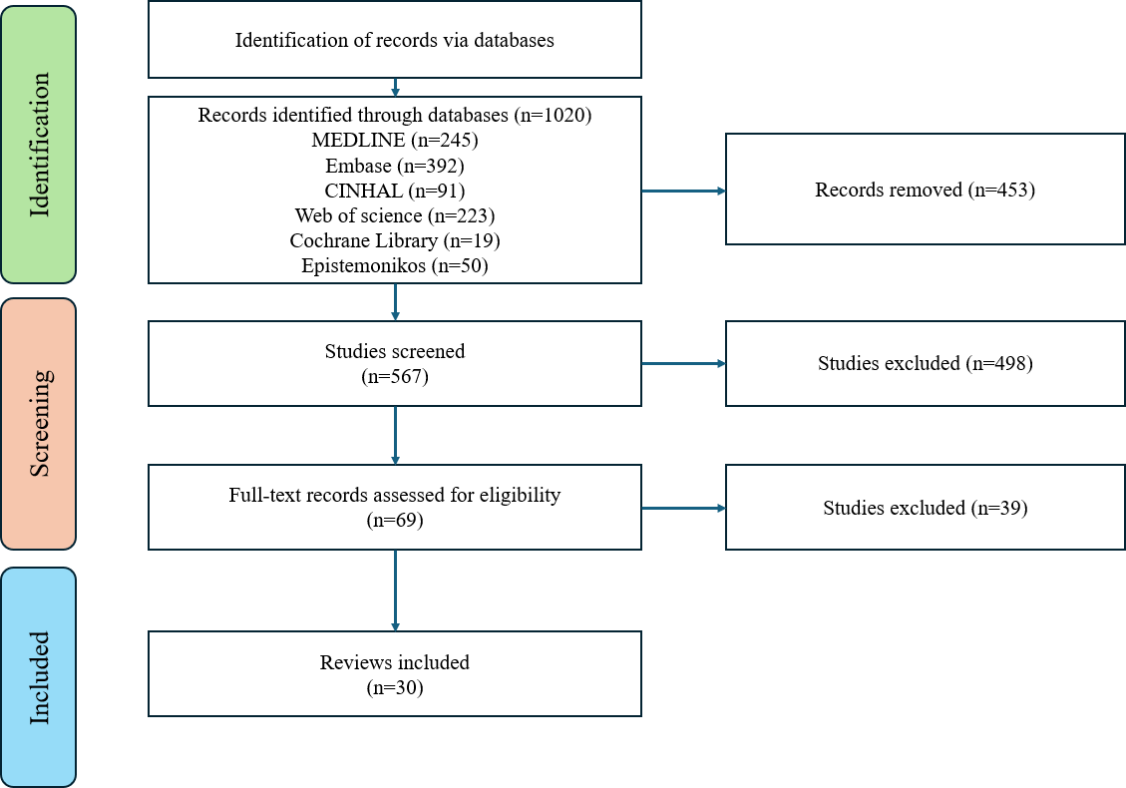
Preferred Reporting Items for Systematic Reviews and Meta-Analyses flow diagram.

The search results yielded a total of 1020 articles. After the removal of duplicates in Covidence (5 duplicate citations identified manually and 448 duplicate citations automatically identified) [36], 567 articles were eligible for title and abstract screening. After excluding nonrelevant studies, a full-text review of 69 studies was conducted, of which 30 SRs met our inclusion criteria [2-4,13,21,48-72]. A flow diagram of study screening and selection procedures is illustrated in the PRISMA flow diagram (Figure 1), with reasons for exclusion of 39 SRs (Multimedia Appendix 2).

### Study Characteristics

We included 30 SRs published between 2016 and 2024 in English (29/30, 97%) [2-4,13,21,48-64,66-72] and French (1/30, 3%) [65]. The number of studies within these SRs ranged from 2 to 39. The number of participants ranged from 130 to 7913, which included patients such as children (9/30, 30%), adolescents (7/30, 23%), adults (10/30, 33%) and elderly (3/30, 10%), and OHCPs (general dentists, dental specialists, dental students, dental hygienists; 4/30, 13%). The countries where the SRs were conducted were Australia (3/30, 10%) [3,13,60], Brazil (7/30, 23%) [2,50,53,59,61,67,69], Canada (1/30, 3%) [49], Chile (2/30, 7%) [49,64], China (1/30, 3%) [70], Colombia (2/30, 7%) [21,58], France (1/30, 3%) [65], Hong Kong (1/30, 3%) [54], Hungary (1/30, 3%) [55], India (1/30, 3%) [68], Italy (2/30, 7%) [4,57], Malaysia (1/30, 3%) [52], Saudi Arabia (1/30, 3%) [72], South Korea (1/30, 3%) [62], the United Kingdom (3/30, 10%) [51,56,71], and the United States of America (3/30, 10%) [48,63,66]. A total of 22 SRs (73 %) [2,3,13,21,48-51,53-55,58-66,68,70] included primary studies conducted in both developing and developed countries, while 2 SRs (7%) [57,67] did not mention the countries of origin of primary studies. The most common study designs of primary studies were randomized controlled trials (15/30, 50%) [2,3,13,48,51,53,54,56,59,62-65,70,72], cross-sectional studies (14/30, 47%) [3,4,48-50,52,55,58-61,64,65,71], and nonrandomized trials (8/30, 27%) [13,49,54,58,63,64,70,72]. Among included SRs, 5 exclusively included RCTs (5/30, 17%) [2,51,53,56,62], whereas 3 SRs included studies on cost analysis (3/30, 10%) [4,13,49], and 3 assessed the impact of teledentistry during the COVID-19 pandemic (3/30, 10%) [52,57,61]. .

In total, 18 SRs (60%) had registered their protocols in PROSPERO (17/30, 57%) [4,49,51-57,59,62-66,69,72] or the Center for Open Science (1/30, 3%) [61]. Among the SRs, 2 did not report information about conflicts of interest (2/30, 7%) [21,53]. A total of 17 SRs (57%) reported that they had received funding to conduct the review [3,4,13,49-52,54,57,59,60,64,66-68,70,71], while 7 did not report information on funding (23%) [2,48,53,61,62,65,72]. Also, among the 30 SRs, 19 reviews (63%) conducted only qualitative synthesis [3,4,13,21,48-50,56-62,65,66,70-72], and 11 reviews (37%) conducted both qualitative and quantitative synthesis [2,51-55,63,64,67-69].

The main domains of dentistry in the included SRs were orthodontics (14/30, 47%) [2,3,19,48,51,53,56,57,62-67], oral medicine (7/30; 23%) [3,13,21,48,49,55,72], and pediatric dentistry (6/30; 20%) [3,19,21,48,60,64]. Excepted orthodontic treatments, teledentistry-related clinical outcomes included those related to periodontal diseases (10/30, 33%) [2,3,51,53,56,57,62-64,66], dental caries (8/30, 27%) [13,21,48,51,55,59,60,68], oral cancer (3/30, 10%) [49,61,69], and health-related knowledge, attitudes, and practices in oral health (11/30, 37%) [2,13,50-52,54,56,58,63,64,70]. In addition, diagnostic accuracy, which included validity and reliability of teledentistry, was assessed in some SRs (8/30; 27%) [3,4,13,48,49,55,58,69]. Half of the SRs evaluated both synchronous and asynchronous modalities of teledentistry (15/30, 50%) [3,4,13,21,49,50,55,57-59,61,64,65,71,72]. The major teledentistry applications were teleconsultation (13/30, 43%) [3,4,13,21,48-50,55,58,61,65,68,71], telediagnosis (9/30, 30%) [4,13,48,55,59,60,68,69,72], telemonitoring or referrals (6/30, 20%) [13,55,61,65-67], and teletriage and telescreening (5/30, 17%) [4,13,21,48,49]

The type of digital technologies mentioned in the SRs were smartphones, intraoral cameras, DSLR cameras, tablets and computers (25/30, 83%) [2,3,13,21,48,51,53-61,63-72]. The most common modes of communication and data transmission were email, text messaging, and applications such as Zoom (Zoom Communications), Telegram, WhatsApp (Meta), WeChat (Tencent), YouTube (Google), and Instagram (Meta; 18/30, 60%) [2,3,13,21,48,51,53-58,60-62,64-66]. The characteristics of the 30 SRs and SR-MAs included in this review are summarized in Multimedia Appendix 3.

### Quality of Systematic Reviews

**Figure 2.**
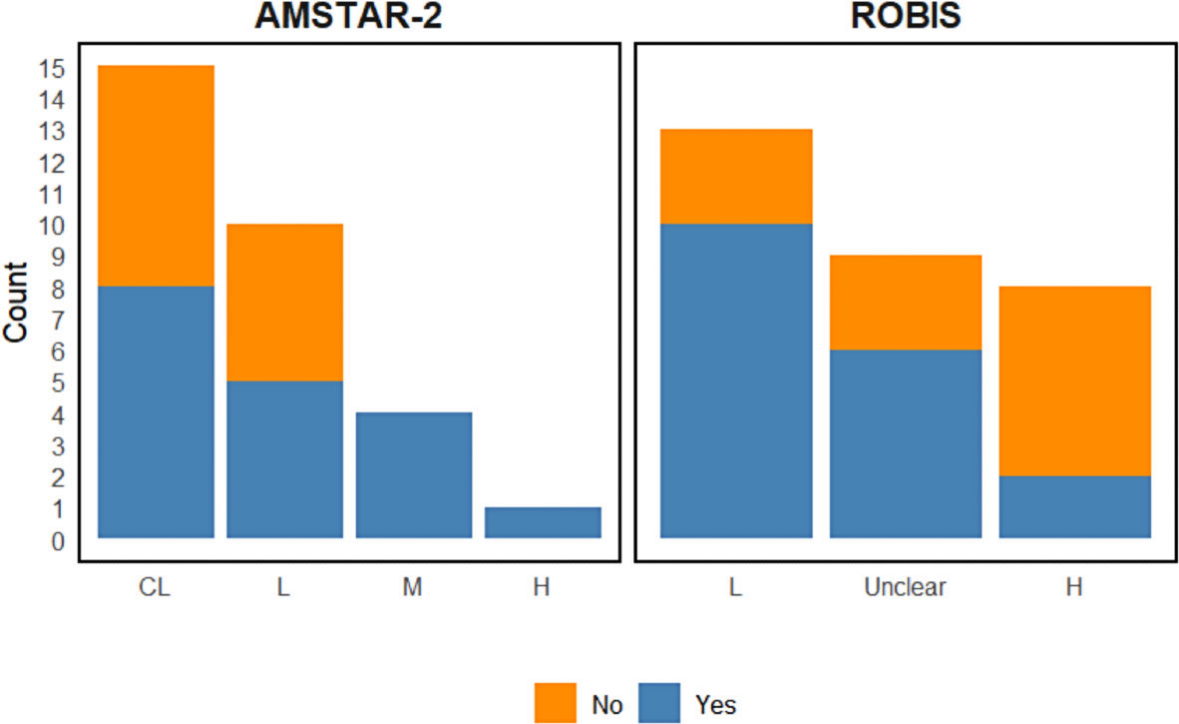
Comparison of A MeaSurement Tool to Assess Systematic Reviews-2 and Risk Of Bias In Systematic reviews ratings based on the published protocol. AMSTAR: A MeaSurement Tool to Assess Systematic Reviews-2; ROBIS: Risk Of Bias In Systematic reviews.

Most SRs (15/19; 79%), including those that performed meta-analyses (10/11; 91%), were rated as “critically low” to “low” quality according to AMSTAR 2 scores. However, ROBIS scores indicated a smaller number of SRs (8/19; 42% and 7/19; 37%), including SR-MAs (2/11; 18.2%), had a “high or unclear” overall risk of bias. The AMSTAR-2 and ROBIS scores are presented in Multimedia Appendices 5 and 6, respectively. SRs with a published or registered protocol demonstrated a better quality overall (Tables1  2 and Figure 2).

**Table 1.** Risk of Bias in Systematic Reviews and a Measurement Tool to Assess Systematic Reviews-2 Rating for Included Systematic Reviews.

Study	ROBIS^a^	AMSTAR 2^b^	Published protocol
[Bibr R72]Abdul et al [72]	Unclear	Critically low	Yes
[Bibr R48]Alabdullah and Daniel [48]	High	Low	No
[Bibr R63]Al-Moghrabi et al [Bibr R63][63]	Low	Critically low	Yes
[Bibr R5]Aquilanti et al [4]	Unclear	Critically low	Yes
[Bibr R71]Bhamra et al [71]	Unclear	Low	No
BöhmdaCosta et al [Bibr R50][50]	High	Critically low	No
[Bibr R70]Chau et al [70]	Low	Critically low	No
[Bibr R62]Choi et al [62]	Low	Moderate	Yes
[Bibr R61]da Silva et al [61]	Unclear	Low	Yes
de Lima et al [69][Bibr R69]	Unclear	Critically low	Yes
[Bibr R49]Emami et al [49]	Low	Moderate	Yes
[Bibr R60]Estai et al [60]	High	Low	No
Estai et al [13][Bibr R14]	High	Low	No
Fernandez et al [Bibr R64][64]	Low	Low	Yes
[Bibr R59]Flores et al [59]	Unclear	Low	Yes
Fortish-Mesa and Hoyos [58][Bibr R58]	High	Critically low	No
[Bibr R4]Irving et al [3]	Unclear	Low	No

a ROBIS- Risk Of Bias In Systematic reviews.

b AMSTAR 2- A MeaSurement Tool to Assess Systematic Reviews-2.

**Table 2.** Risk Of Bias In Systematic reviews and A MeaSurement Tool to Assess Systematic Reviews-2 rating for included systematic reviews, Cont.

Authors	ROBIS^a^	AMSTAR 2^b^	Published protocol
[Bibr R53]Lima et al^c^ [Bibr R53][53]	Low	Low	Yes
[Bibr R52]Lin et al^c^ [52][Bibr R52]	Low	High	Yes
[Bibr R51]Mohammed et al^c^ [51][Bibr R51]	Low	Low	Yes
[Bibr R68]Priyank et al^c^ [Bibr R68][68]	Unclear	Critically low	No
[Bibr R65]Rouanet et al [65]	High	Critically low	Yes
[Bibr R57]Saccomanno et al [57]	High	Critically low	Yes
[Bibr R66]Sangalli et al [66]	Low	Moderate	Yes
[Bibr R56]Sharif et al [56]	Unclear	Moderate	Yes
[Bibr R3]Toniazzo et al^c^ [Bibr R3][2]	Low	Critically low	No
[Bibr R67]Torres et al^c^ [Bibr R67][67]	Low	Critically low	No
[Bibr R22]Troconis et al [21]	High	Critically low	No
[Bibr R55]Uhrin et al^c^ [[Bibr R55][55]	Low	Critically low	Yes
[Bibr R54]Wang et al^c^ [Bibr R54][54]	Low	Low	Yes

a ROBIS- Risk Of Bias In Systematic reviews.

b AMSTAR- A MeaSurement Tool to Assess Systematic Reviews-2.

c Systematic review without meta-analysis.

### Certainty of Evidence

The certainty of evidence for the outcomes was assessed using GRADE in 10 included SRs (10/30, 33%). However, the diverse findings led to inconclusive certainty of evidence regarding the different outcomes. GRADE was found to be very low (2/10, 20%) [2,63], low (1/10, 10%) [63], moderate (3/10, 30%) [53,56,64], and high (1/10, 10%) [53] for gingival index. It was very low (3/10, 30%) [2,63,64], low (1/10, 10%) [64], moderate (2/10, 20%) [51,56], and high (long-term; 2/10, 20%) [51,53] for plaque index. Regarding white spot lesions, GRADE was moderate (1/10, 10%) [64] and high (2/10, 20%) [51,53]. One SR reported a very low GRADE for 4 outcomes (knowledge, feeding oral hygiene status, attitude, and tooth cleaning) and a low GRADE for caries status (1/10, 10%) [54]. GRADE was low to moderate for accuracy in detecting oral premalignant lesions (1/10, 10%) [55], and very low for the detection of malignant oral lesions (1/10, 10%) [69]. For measuring and monitoring maxillary expansion outcome, GRADE was reported to be very low (1/10, 10%) [67]. Two SRs used the Oxford Centre for Evidence-based Medicine level of evidence (OCEBM) with the score mostly at level 4 and 3b (1/10, 10%) [49], and Level 3 (1/10, 10%) [52]. The strength of evidence for the diagnostic accuracy of teledentistry with the Jovell and Navarro-Rubio classification was Category VII (1/10, 10%) [60], corresponding to a fair strength of evidence.

### Heterogeneity

Among the 19 SRs which did not conduct the meta-analysis, only 11 SRs explicitly (58%) reported “high heterogeneity” as the reason [3,4,13,49,56,57,59,60,62,65,66]. High heterogeneity was reported in 1 SR (1/19, 5%) and 10 SR-MAs (10/11, 91%) [2,51-55,63,64,67-69]. Heterogeneity was highlighted among primary studies of SR-MAs, for plaque scores (92%) and gingival scores (97%; 1/11, 9%) [64], knowledge, awareness, and practices of teledentistry among OHCPs (>90%; 1/11, 9%) [52], the detection of oral lesions (2/11, 18%) [55,69], and the diagnostic accuracy for the detection of dental caries (1/11, 9%) [68]. There was no heterogeneity (*I*^2^=0%) among studies for dental caries (1/11, 9%) [64], the diagnosis of oral premalignant lesions and oral cancer (1/11, 9%) [55], and white spot lesions (1/11, 9%) [64]. One SR-MA on plaque, gingiva,l and bleeding index did not provide *I^2^* statistics (1/11, 9%) [63]. Among SR-MAs, 2 reviews (2/11, 18%) [51,53] conducted subgroup analysis at 2 time periods (short term: 3 months, and long term: 3‐6 months) for plaque and gingival scores, and 1 review on white spot lesions (1/11, 9%) [53]. In the short term, heterogeneity for plaque scores differed between the 2 SRs as they reported high (92%) [53] and low (24%) [51] values, respectively. There was very low heterogeneity (*I^2^*=0%) in both the short- and long-term for white spot lesions (1/11, 9%) [53]. Another SR-MA reported high heterogeneity (>95%, 1/11; 9%) for both plaque scores and gingival bleeding, even with a subgroup analysis for age [2].

### Overlap of Studies

The CA of the primary studies included in all the SRs was moderate (5.56%) while the CCA was 2.3%, suggesting a slight overlap. For the primary studies pooled studies within the 11 SR-MAs, the CA was high (14.29%) compared with a moderate CCA (5.71%). There was a very high overlap of the primary studies among SR-MAs that assessed clinical outcomes. The CA and CCA of primary studies among 5 SR-MAs for plaque index (5/30, 17%) [2,51,53,63,64], gingival index (5/30, 17%) [2,51,53,63,64], and white spot lesions (3/30, 10%) [51,53,64], respectively (38.3% and 28.9% vs 43.8% and 28.8% vs 83.3% and 75%). We presented the graphical visual of the overlap (GROOVE) for the 30 SRs (Multimedia Appendix 6). The GROOVE for the SR-MAs is provided in Multimedia Appendix 7. The GROOVE for each of the clinical outcomes (gingival index, plaque index, and white spot lesions) is provided in MultimediaAppendices 8^10^.

### Research Findings

The SRs’ findings, including patients’ and OHCPs’ indicators and outcomes, are grouped under domains of quality of care [30-32] and according to authors’ reports as follows: (1) timely and equitable access to teledentistry; (2) patient-centered care including barriers and enablers to implementing teledentistry; (3) patient safety (eg, privacy); (4) efficiency (eg, costs); and (5) effectiveness in improving oral health, including experiences, clinical effectiveness, and accuracy of teledentistry.

#### Access to Care, Timely and Equity Toward Teledentistry

A total of 9 SRs reported some aspects related to access to care, timely and equity toward teledentistry (9/30, 30%) [3,13,21,49,50,57,61,64,65]. Teledentistry can be a valuable tool for overcoming public health challenges related to poor access to oral health services and oral health inequities (1/9, 11%) [3] as well as in the prevention of oral diseases and in oral health promotion (1/9, 11%) [64]. As a viable option, teledentistry offers several advantages such as access to dental care (3/9, 33%) [3,50,61], especially during the COVID-19 pandemic and for underserved communities (6/9, 67%) [3,13,21,49,50,57]. Teledentistry applications include long-distance consultations, remote dental examinations, screening of digital images and radiographs, triage, early detection of diseases, telediagnosis, and access to general dentists and dental specialists (1/9, 11%) [3]. Voice calls and smartphone apps such as WhatsApp and Messenger (Meta) were the most common modes of communication between patients and dental staff (2/9, 22%) [57,65]. In addition, 1 review reported that teledentistry supports oral health equity by reducing care costs and increasing awareness through technology among various people (1/9, 11%) [50].

#### Patient-Centered Care With Teledentistry

In total, 14 SRs highlighted the patient-centeredness of teledentistry (14/30, 47%) [50,54,57-59,61,62,65,70,72], including 7 reviews focusing on the determinants of teledentistry adoption (7/14, 50%) [3,4,49,50,52,58,61] Teledentistry was reported as an effective approach in the diagnosis, management, and treatment of oral diseases (2/14, 14%) [58,59]. It has a positive impact on oral health knowledge, attitudes, acceptance, and behavior change (3/14, 21%) [54,57,70]. In addition, it enhances the continuity of care [57], communication between patient and clinicians or between clinicians themselves, improving the quality of care and patients’ outcomes (3/14, 21%) [50,61,65]. Teledentistry also assists clinicians in managing orthodontic emergencies and completing orthodontic treatment (2/14, 14%) [57,61]. In addition, it contributes to a positive experience in managing temporomandibular symptoms (1/14, 7%) [72]. Daily text messaging was found to significantly reduce the intensity of self-reported pain among patients (1/14, 7%) [62].

Factors related to patients/caregivers and OHCPs, as well as to contextual and structural levels, influence patient-centered teledentistry, its applicability and its effectiveness (6/14, 43%) [3,4,50,52,58,61]. The factors related to patients included familiarity and the ease of using digital technologies (1/14, 7%) [61]. For OHCPs, several factors (eg, fear of making an inaccurate diagnosis, concerns about increased costs, insufficient financial reimbursement, and lack of training and skills) could influence teledentistry implementation positively or negatively (3/14, 21%) [49,58,61]. Furthermore, OHCPs’ education level and years of experience may influence its implementation and its perception (1/14, 7%) [52]. The acceptance of clinicians and patients/caregivers is crucial to adopting teledentistry (3/14, 21%) [3,4,61]. One SR (7%) [52] reported limited knowledge (1/14, 57.9%, 95% CI 46-69.9) and poor practice in teledentistry (35.8%, 95% CI 14.8-56.8) among OHCPs during the COVID-19 pandemic despite high level of awareness (70.4%, 95% CI 64.3-76.5), and positive attitude (72.5%, 95% CI 60.7-84.3). Tele-education, teleassistance, and training through workshops, lectures, or seminars could help to improve the successful implementation of teledentistry (2/14, 14%) [50,52]. At the structural and contextual levels, internet access (1/14, 7%) [52], available technologies and their lower costs (1/14, 7%) [3], support from information technology personnel (2/14, 14%) [4,52], and governmental support (2/14, 14%) [3,50] were crucial for the sustainability of teledentistry. On the other hand, the variability of infrastructure between countries (particularly between developed and developing countries), conflicting legislation, inadequate financial remuneration, disparities in rural regions, and lack of guidelines were cited as factors hindering teledentistry applications (2/14, 14%) [52,61].

#### Efficiency of Teledentistry

In total, 11 SRs reported on some aspects of the efficiency of teledentistry (11/30, 37%) [3,4,13,21,49-51,62,65-67]. Teledentistry is cost-effective due to a reduction in waiting lists and unnecessary travels (3/11, 27%) [3,4,62] for vulnerable people (3/11, 27%) [4,21,49] For instance, teledentistry saves an average of 50 minutes of travel time per visit (1/11, 9%) [65], and preventing or reducing the loss of productivity among working patients (1/11, 9%) [13]. In addition, teledentistry may reduce the average waiting time for general and dental specialized care (2/11, 18%) [3,13], for instance for patients (3.33 days vs 28 days) and the cancellation rate on the day of surgery (7.8% vs 8.85%), as compared with conventional oral health delivery (1/11, 9%) [49], and in-office visits (1/11, 9%) [66]. This allows time and financial resources to be redirected to patients with higher oral health risks (1/11, 9%) [50]. One SR reported a significant reduction in the number of in-person appointments (mean difference=−2.75 [95% CI −3.95,‐1.55]) and a shorter time to start orthodontic treatment (mean difference=−1.21 [95% −2.35,‐0.08]) with teledentistry monitoring compared to face-to-face monitoring (1/11, 9%) [67].

Asynchronous teledentistry has shown lower costs than in-person or real-time consultations (2/11, 18%) [13,49] due to a reduction in the costs of travel for OHCPs (1/11, 9%) [49] and lower training costs (1/11, 9%) [4]. However, asynchronous approaches could require more time compared with synchronous approaches (20 min for store-and-forward vs 15 min for real-time examinations; 1/11, 9%) [4]. Some conflicting results were reported on the impacts of teleorthodontics, regarding the duration of treatment (4/11, 36%) [51,62,65,66], and the number of emergency appointments (1/11, 9%) [66]. Additional costs for health organizations and society are reported for teledentistry compared with outreach visits by dental specialists in remote communities (1/11, 9%) [49], while teleconsultation reduces annual patient costs by 69% (eg, transportation, accommodation, and lost productivity; 1/11, 9%) [49].

#### Patients’ Safety Related to Teledentistry

Several SRs reported the potential implications of teledentistry for patients’ safety (8/30, 27%) [3,4,50,52,56,57,61,72]. Teledentistry could be effective in reducing the risk of cross-infection, as evidenced during the COVID-19 pandemic (1/8, 12.5%) [61], as well as in alleviating anxiety, fear, and a sense of abandonment during dental treatments (1/8, 12.5%) [57]. Some SRs raised teledentistry issues related to patient privacy and confidentiality (5/8, 63%) [3,50,52,61,72]. Two authors reported no adverse events with teledentistry (2/8, 25%) [4,56].

#### Effectiveness With Teledentistry

A total of 22 SRs focused on the effectiveness of teledentistry (22/30, 73%) [2,4,48-51,53-57,59-65,68-70,21]. We will present three components of this domain of quality of care as follows: patients and OHCPs' experiences, clinical outcomes, and accuracy with teledentistry,

##### Patients and OHCPs’ Experiences With Teledentistry

We identified 8 SRs that reported patient satisfaction with teledentistry (8/22, 36%) [4,49,50,57,61,64,65,62] such as orthodontics patients (1/8, 12.5%) [62], elderly residents and their families (1/8, 12.5%) [4], and patients with oral cancer (1/8, 12.5%) [61]. Teledentistry was appreciated by both clinicians and patients (3/8, 38%) [50,57,65], and most of them have expressed optimism and satisfaction with its integration into current dental practices (1/8, 12.5%) [50]. For instance, a range of 63%‐78% of patients living in rural and remote areas were satisfied with e-oral health care interventions (1/8,12.5%) [49]. The high satisfaction rate regarding teledentistry was mainly attributed to fewer hospital visits, less traveling time, a better understanding of oral health care needs and self-management, cost savings on transportation, effective communication, and shorter waiting periods (4/8, 50%) [4,49,61,64]. Patients expressed better compliance (1/8, 12.5%) [61], along with a decreased likelihood of missing appointments (RR 0.39; 95% CI 0.22-0.70; 1/8, 12.5%) [51], an adherence to oral hygiene (4/8, 50%) [51,53,56,64] and appointments (1/8, 12,05%) [51] with teledentistry. Some patients highlighted the potential of teledentistry in enhancing their oral health (1/8, 12.5%) [65], and improving their well-being and quality of life (1/8, 12.5%) [61]. Patients preferred videophones over telephone examinations (80%), because of their perceived ease of use and the willingness to recommend it to other people (1/8,12. 5%) [61].

##### Clinical Outcomes With Teledentistry

In total, 9 SRs (9/22, 41%) [2,51,53,54,56,62-64,70] assessed the effectiveness of teledentistry in improving oral health. Among these reviews, reviews (6/9, 67%) [2,51,53,54,63,64] pooled the results of the studies and conducted meta-analyses. The most assessed outcomes were plaque and gingival scores, white spot lesions, and dental caries among orthodontic patients. Significant improvements were found in the plaque index scores and gingival index (6/9, 67%) [2,51,53,54,63,64] along with a reduction in white spot lesions (6/21, 29%) [2,51,53,54,63,64]. One study highlighted the positive impact of reminders, as a reduction in bracket failure (11.8% vs 16.1 %; 1/9, 11%) [51]. Conversely, another study found that reminders had no significant effect on bleeding on probing (SMD=−0.22, 95% CI −0.5 to 0.05; 1/9, 11%) [63]. mHealth was found to improve oral health (2/9, 22%) [2,70], and reduce the frequency of self-reported pain among orthodontic patients (1/9, 11%) [62]. While it improved parents’ knowledge about children’s oral health, it did not improve their children’s oral health (1/9, 11%) [54].

##### Accuracy of Teledentistry

A total of 9 SRs (9/22, 41%) [4,48,49,55,59,60,68,69,21] found teledentistry to be as reliable as in-person clinical examination, screening and diagnosis of oral diseases, the detection of root canals, caries assessment, referrals and teleconsultations, and the management of oral infections. Authors reported the potential of teledentistry for diagnosis and treatment planning (2/9, 22%) [21,48,49], patient triage (1/9, 11%) [49], particularly for populations in rural regions (1/9, 5%) [21], and older adults in nursing homes (1/9, 11%) [8].

The sensitivity and specificity of the teledentistry-based assessments showed significant agreement with clinical consultation (1/0, 5%) [59] and clinicopathological examination (1/9, 11%) [49]. For instance, teledentistry demonstrated its potential and reliability in the detection of oral lesions (sensitivity: 0.92, CI 0.59-0.99; specificity: 0.93, CI 0.17-1.00; 1/9, 11%) [55]. pre-malignant oral lesions (sensitivity and specificity values of 0.93 [0.91‐0.95] and 0.98 [0.97‐0.99], respectively) (1/9, 11%) [69]. caries lesions (sensitivity ranged from 43% to 100% and specificity from 52% to 100%) (1/9, 11%) [60] and for differential diagnosis of oral lesions (sensitivity 0.92, CI 0.84-0.97; specificity 0.99, CI 0.95-1.00; 1/9, 11%) [55]. Photographic methods (1/9, 11%) [60] using smartphones or intraoral cameras (1/9, 11%) [68] showed comparable results for caries assessment. Teledentistry tools (email, free chat applications, cloud-based storage applications, imaging, etc.) were reliable options for replacing face-to-face dental visits (1/9, 11%) [55]. However, the validity of teledentistry could be influenced by the access to patient information and the experience with digital technologies of dental professionals (1/9,11%) [48].

## Discussion

### Principal Findings

The aim of this overview was to summarize evidence from SRs with or without meta-analysis on the impacts of teledentistry, providing a comprehensive insight to inform clinical and policy decision-making. Our overview covered all the domains of quality to various degrees, with the domains of effectiveness, patient-centered care, efficiency, and access to teledentistry being the most studied. Similarly, a recent mapping review on digital health and quality of health care [5], and an umbrella review on telemedicine [73] also highlighted the greater number of studies on its clinical effectiveness, with the equity domain addressed by few SRs. This result corroborates those of a recent SR on the quality of care in dentistry, highlighting poor research on various dimensions of quality in primary dental care, such as patient safety and equity in dentistry [74,75]. Our results suggest that teledentistry may be a reliable alternative to usual care [4,48,49,55,59,60,68,69] for clinical examinations, triage, screening, diagnosis, and treatment planning, and reducing inappropriate referrals, particularly for populations living in rural regions [21] and older adults living in nursing homes [8]. Teleconsultation, triage, telediagnosis, and telemonitoring were the most common activities within the current field of teledentistry. The sensitivity and specificity of the teledentistry-based assessments showed significant agreement with clinical consultation [59] and with clinicopathological examination [49]. Therefore, teledentistry is effective in caries assessment, root canal detection, referrals, and managing oral infections, resulting in a greater number of patients treated for malignant oral disorders [55]. Photographic methods [60] with smartphones or intraoral cameras [68] showed comparable results for caries assessment. Tools like smartphones, intraoral cameras, email, and cloud-based applications offer comparable results to face-to-face oral health visits for procedures [55], while their validity may depend on access to patient information and the experience of dental professionals [48]. Despite asynchronous teledentistry generally incurring lower costs compared with in-person or real-time consultations [13,49], it may require more time than synchronous approaches [4].

Based on the structural domain approach, and in line with the Quintuple Aim’s 5 overarching goals [76] to redesign health care delivery systems, the findings of this overview indicated teledentistry’s potential to enhance patients’ experience of oral health care delivery; the health of populations; improve health care providers’ well-being and health; and reduce costs while contributing to improving health equity. The impacts of teledentistry on patient-reported outcomes (eg, self-reported pain, self-management, and self-performed daily oral health hygiene) and experiences were highlighted through the domains of effectiveness and patient-centered care. Teledentistry led to improved clinical outcomes, patients’ and OHCPs’ satisfaction, and enhanced communication [4,49,61] Furthermore, teledentistry may contribute to improving clinical outcomes (eg, plaque scores, gingival scores, white spot lesions, and dental caries). It had the potential to reduce oral health care inequities, ensuring timely oral health services, improved care experiences, and better quality of life [3,4,21,49,50]. In dental practice, it increased consultations, decreased unnecessary referrals, and redirected resources to high-risk patients [4,50,55,62]. In addition, it helped prevent cross-infections during the COVID-19 pandemic, as well as the anxiety and the fear for certain patients. Teledentistry lowered costs, minimized treatment delays [3,13], and productivity loss among working patients [13]. The promising cost-effectiveness of teledentistry was reported mostly for asynchronous modalities [4,49], mainly due to reduced travel costs for oral health professionals and dental assistants, as well as savings on transportation and accommodation, along with cost savings related to staff salaries for school dental screening programs [49]. Within dentistry, staff travel for work purposes and commuting to work (33.4%) and patient travel to dental practices (31.1%) are often the most carbon-intensive activities [77]. Furthermore, unnecessary travels may lead to reduced energy consumption, greenhouse gas emissions, and waste production [78]. Given teledentistry was reported to reduce travel from both patients and OHCPs, we could anticipate environmental benefits of teledentistry (eg, used computer, internet, and telephone), as telemedicine [79,80], and more sustainable delivery of oral health care [81,82].

Despite these benefits, several interlinked factors at macro, meso, and micro levels influence the successful implementation of teledentistry. At the macro level, regulatory policies and procedures [4], the lack of remuneration, legal issues including licensure, jurisdiction, malpractice, and privacy [50], and the availability of infrastructures and resources in health care systems will influence the effectiveness and broad applicability of teledentistry. At the meso level, teledentistry requires additional expertise and equipment to maintain high-quality care within the dental environment. Notably, the influence of contextual factors, such as the dental environment, and their interactions with individual and systemic elements may play a critical role in shaping OHCPs’ competencies and performance in adopting teledentistry. At the micro level, lack of knowledge and skills with the use of teledentistry [52], unfamiliarity regarding the functional use of digital devices, anxiety toward technology [52], are important factors. The diversity of these multilevel factors can significantly affect the sustained adoption of teledentistry.

Several SRs had a critically low to low methodological quality scoring and a high risk of bias, which could influence the robustness of the results. In general, there were many studies with a low risk of bias in comparison with the number of studies with a high methodological quality. One potential explanation for the very low proportion of studies with high methodological quality scoring is that nearly 40% of SRs had not registered their protocols, leading to unnecessary duplicates and wastage in research. According to other studies in the literature on differences between the AMSTAR 2 and ROBIS ratings [41,83,84], this discrepancy between ROBIS and AMSTAR2 highlights the nuance between these 2 tools, emphasizing their relevance in evaluating the conduct of reviews, the way authors report their results, and the importance of publishing the protocol of SRs [85]. Despite this difference, the GRADE level of evidence among SR-MAs varied for the same outcomes. This discordance could be due to the difference in inclusion criteria and the time range of measures (3 vs 6 vs 12 vs 18 months). Assessing the degree of overlap in overviews of SRs is important, given that they can generate valuable and reliable information to guide policies and practices [47,86]. Similar to this overview wherein the overall CCA was 2.3%, a low degree of overlap is usually something to be expected for a broad-scope overview of systematic reviews [86]. This slight overlap of primary studies resulted from the inclusion of SRs that evaluated diverse outcomes. However, when focusing on individual outcomes within SR-MAs, a significant overlap was observed. Given these observations, on the variability in the certainty of evidence and heterogeneity reported in several of the included SRs, the results should be interpreted with caution. Repeated inclusion of primary studies can lead to a redundancy of the evidence, particularly in case of a high overlap among SR-MAs reporting the same outcomes.

### Strengths and Limitations of Our Overview

Although there are previous overviews of teledentistry in relation to some specific health conditions [24-26], to our knowledge, this is the first comprehensive overview that compiles results on teledentistry regarding access to and quality of care, while incorporating the assessment of both methodological quality and risk of bias of the included SRs. Our overview has several strengths. First, it used a comprehensive search strategy in many databases, without any publication, date, and language restrictions, to identify and summarize the evidence on teledentistry. We identified many gaps and priority areas for future research. Second, we used 2 robust tools to assess the quality of included SRs. Two reviewers were involved in the screening, the data extraction, and the quality assessments. In addition, the protocol was registered and published. Third, this overview is comprehensive, includes multiple domains, and assesses multiple outcomes regarding teledentistry.

Despite the use of rigorous methods, some limitations still exist. First, we did not search for gray literature, potentially limiting the representativeness of our findings compared with all relevant work in the field. However, traditional databases, such as Embase and Web of Science, already index gray literature, including conference proceedings, preprints, and dissertations. In addition, gray literature is often not peer-reviewed. According to our inclusion criteria, additional searches for gray literature were deemed unnecessary and unlikely to enhance our search strategy and/or findings. Second, we did not retrieve data from the primary studies included in our review, notably for the certainty of evidence; however, reanalysis of primary data and conducting a new meta-analysis is not often required in an overview [87]. Third, some included SRs presented various time horizons, while other SRs and SR-MAs did not provide this information. As a result, we are unable to differentiate between short-term and long-term outcomes and thus cannot conduct an in-depth investigation of teledentistry sustainability over time. Fourth, beyond the primary studies overlap, our overview highlights the overrepresentation of some countries (mostly Brazil, the United Kingdom, and the United States), specialties (eg, orthodontic care), populations (eg, adolescents), outcomes (periodontal parameters), and modalities (asynchronous teledentistry). Together, these may have an impact on generalizability, limiting the applicability of our findings, for instance, to other countries where policy, awareness, interest, and infrastructure for teledentistry and health care resources may vary. Fifth, the heterogeneity of the systematic reviews and the varying certainty levels of individual clinical outcomes may influence the quality of evidence. Furthermore, subgroup analyses were not performed, as they were not feasible due to the substantial variability across the studies. Subgroup analysis should be planned early in the study design and conducted when feasible to generate meaningful insights regarding their direct impact on a topic [88]. Sixth, while a calibration exercise was performed, some quality assessment errors may have occurred given the challenges in using both ROBIS and AMSTAR-2. Despite these limitations, this comprehensive overview gives valuable insights, along with an in-depth understanding of the body of evidence on teledentistry.

### Implications

Overviews systematically compile, appraise, and synthesize findings from related SRs to support informed health care decision-making. They are valuable tools for clinicians, policymakers, and guideline developers, by enhancing the access to scientific evidence. This overview on teledentistry has significant implications for policy as it has consolidated evidence from multiple sources, offering a comprehensive synthesis to make informed decisions based on reliable insights. In addition, our findings support the development of equitable, evidence-based policies to improve access and the quality of oral care. Finally, the study findings highlight knowledge gaps, guiding future research priorities and resource allocation toward digital health solutions and teledentistry.

For oral health care professionals (OHCPs), this overview provides a robust and up-to-date summary of evidence on teledentistry. It may increase OHCPs’ awareness of existing evidence on teledentistry, inform their clinical decision-making, and enable them to deliver high-quality oral care. By reducing uncertainty, our findings also foster clinicians’ confidence to integrate teledentistry into their practices. Furthermore, our overview identifies areas where evidence is weak or lacking, guiding clinicians to exercise caution when necessary.

Finally, this overview contributes to evidence-based dental education and training by providing educators with the latest knowledge on teledentistry. This can enhance dental curricula, support professional development, and prepare students and other learners to effectively use teledentistry through research-driven learning and education. By equipping future OHCPs with the necessary skills and knowledge, our findings foster innovation and readiness for evolving teledentistry interventions.

### Future Research

This overview addressed the critical need for a comprehensive understanding of the quality of care with teledentistry. Authors of SRs reported limitations related to the small number of studies, language restrictions, heterogeneity, low certainty of evidence, different follow-up periods, low-quality studies, nontransparent reporting of interventions, and limited generalizability, which restricted the robustness of their findings. They also highlighted the priorities for future research. While the overview addresses all domains of quality of care, there remains a scarcity of studies on patient safety and equity in teledentistry. Most of the reviews have involved adolescents and young adults undergoing orthodontic treatments, highlighting the need for evidence on different population groups and cultures, a broader range of settings as well as dental specialties for more comprehensive evidence on teledentistry’s impact. The quality of images captured through devices such as intraoral cameras, DSLR cameras, and smartphone cameras could impact the accuracy of diagnosis, resulting in the need to standardize the tools used in teledentistry to compare data. A limited number of included reviews have used behavior change theories, highlighting the relevance of theories, models, and frameworks in implementation science to improve teledentistry implementation efforts among OHCPs and patients. A small number of studies focus on the environmental impact, despite the escalating threat of climate change and increasing greenhouse gas emissions. In the future, it will also be essential to conduct some studies on equity, patient safety, and the environmental impact of teledentistry in reducing the carbon footprint of oral health care, and in context-specific dental settings. They are crucial within the quality-of-care framework to ensure the sustainability of teledentistry implementation [89]. Furthermore, studies that identify the barriers to teledentistry implementation in diverse geographical locations and health care systems are essential in developing tailored strategies to enhance its adoption and sustainability. Investigating subgroup-specific differences could deepen our understanding of teledentistry’s impacts, guiding evidence-informed practices and policies to support targeted interventions, and ultimately enhancing patients’ outcomes and experiences. Finally, robust and high-quality research is crucial to underscore the potential of teledentistry for maximizing the quality of oral health care and key stakeholders’ outcomes and experiences.

### Conclusions

The findings of this overview highlight the growing body of knowledge on teledentistry. The evidence offers valuable insights for policymakers, researchers, and OHCPs. As a patient-centered, effective, and efficient alternative to in-person oral health care, teledentistry enhances access to care. However, significant gaps remain in understanding teledentistry’s impact on patient safety, equity, and the environment, highlighting the need for further research to ensure the high quality of care delivered through teledentistry. Given the rapid advancements in digital technologies and their global adoption, dental regulatory bodies need to establish clear guidelines to optimize the implementation and sustainability of teledentistry across diverse settings, including rural, urban, private, and public dental care environments. Furthermore, the number of duplicate studies and the generally low or very low quality of most included SRs underscores the urgent need for robust research on teledentistry. Addressing these gaps is essential to fully realize the potential of teledentistry in improving health outcomes, ensuring equitable access to care, and providing a solid evidence base to guide clinical practices and inform policy decisions.

## Supplementary material

10.2196/65211 Multimedia Appendix 1 Search strategy.

10.2196/65211 Multimedia Appendix 2 List of excluded studies with reasons.

10.2196/65211 Multimedia Appendix 3 Characteristics of included systematic reviews.

10.2196/65211 Multimedia Appendix 4 AMSTAR 2

10.2196/65211 Multimedia Appendix 5 Risk Of Bias In Systematic Reviews.

10.2196/65211 Multimedia Appendix 6 Graphical Representation of Overlap for Overviews Systematic Reviews.

10.2196/65211 Multimedia Appendix 7 Graphical Representation of Overlap for Overviews systematic reviews – meta-analysis.

10.2196/65211 Multimedia Appendix 8 Graphical Representation of Overlap for Overviews gingival index.

10.2196/65211 Multimedia Appendix 9 Graphical Representation of Overlap for Overviews plaque index.

10.2196/65211 Multimedia Appendix 10 Graphical Representation of Overlap for Overviews white spot lesions.

10.2196/65211 Checklist 1 PRISMA checklist.

## Abbreviations

AMSTAR-2: A Measurement Tool to Assess Systematic Reviews-2
CA: covered area
CCA: Corrected Covered Area
GRADE: Grading of Recommendations, Assessment, Development, and Evaluation
ICT: Information and Communication Technologies
OHCP: Oral Health Care Providers
PRISMA: Preferred Reporting Items for Systematic Review and Meta-Analyses
QUADAS: Quality Assessment of Studies of Diagnostic Accuracy
RCT: Randomized Controlled Trials
ROBIS: Risk of Bias in Systematic Reviews
SR-MAs: Systematic Reviews – Meta-Analysis
SRs: Systematic Reviews
