# Supplementary material for: Teledentistry for Improving Access To, and Quality of Oral Health Care: Overview of Systematic Reviews and Meta-Analyses
Source: J Med Internet Res. 2025 Jul 30;27:e65211. doi: 10.2196/65211 (PMC12334114; doi:10.2196/65211)
Supplement: Multimedia Appendix 1 [file jmir-v27-e65211-s001.docx]

**Database search strategy**

**Medline (OVID)**

**Date of the search:** 07-03-2024

**Database limit:** No database limit has been applied.

| **#** | **Search strategy** | **Results** |
| --- | --- | --- |
| 1 | Teledentistry.ti,ab,kf,kw OR "tele-dentistry".ti,ab,kf,kw |  |
| 2 | Dental Health Services/ OR Dentists/ OR exp Dentistry/ OR exp Mouth Neoplasms/ |  |
| 3 | dental.ti,ab,kf,kw OR dentist*.ti,ab,kf,kw OR orthodonti*.ti,ab,kf,kw OR periodont*.ti,ab,kf,kw  OR prosthodont*.ti,ab,kf,kw OR (oral adj2 (health OR care OR surger* OR Diagnos* OR Hygiene OR Medicine OR lesion?)).ti,ab,kf,kw OR ((oral OR Mouth) adj1 (cancer OR Neoplasm?)).ti,ab,kf,kw |  |
| 4 | 2 OR 3 |  |
| 5 | exp Telemedicine/ OR Mobile Applications/ OR exp Cell Phone/ OR exp Videoconferencing/ |  |
| 6 | Telemedicine.ti,ab,kf,kw OR Telehealth.ti,ab,kf,kw OR Teleconsultation.ti,ab,kf,kw OR telediagnosis.ti,ab,kf,kw OR teletriage.ti,ab,kf,kw OR telemonitoring.ti,ab,kf,kw OR mHealth.ti,ab,kf,kw OR eHealth.ti,ab,kf,kw OR "e-health".ti,ab,kf,kw OR "Mobile Health".ti,ab,kf,kw OR (remote adj2 (telecommunication OR Consultation OR care OR diagnosis)).ti,ab,kf,kw OR (tele adj2 (medicine OR consultation* OR health OR diagnosis OR triage)).ti,ab,kf,kw OR (virtual adj2 (consult* OR care)).ti,ab,kf,kw OR econsult*.ti,ab,kf,kw  OR "e consult*".ti,ab,kf,kw OR ((Video OR Electronic) adj2 Consult*).ti,ab,kf,kw OR "mobile app*".ti,ab,kf,kw OR "cellular phone".ti,ab,kf,kw OR smartphone*.ti,ab,kf,kw OR "text messag*".ti,ab,kf,kw OR "mobile phone".ti,ab,kf,kw OR Videoconferenc*.ti,ab,kf,kw |  |
| 7 | 5 OR 6 |  |
| 8 | Meta-Analysis/ OR Systematic Review/ OR Review/ |  |
| 9 | (scoping adj2 (stud* OR review?)).ti,ab,kf,kw OR (evidence adj2 map*).ti,ab,kf,kw OR "Environmental scan*".ti,ab,kf,kw OR "evidence synthesis".ti,ab,kf,kw OR "systematic map*".ti,ab,kf,kw OR overview?.ti,ab,kf,kw OR "review of review?".ti,ab,kf,kw OR "rapid review?".ti,ab,kf,kw OR (Narrative adj1 (review OR summary OR Synthesis)).ti,ab,kf,kw OR (mixed adj1 (method? OR stud*)).ti,ab,kf,kw OR "Research Synthesis".ti,ab,kf,kw OR "Meta-synthesis".ti,ab,kf,kw OR "Meta-Theory".ti,ab,kf,kw OR "Meta-Study".ti,ab,kf,kw OR "Meta Ethnography".ti,ab,kf,kw OR "Meta-aggregation".ti,ab,kf,kw OR "Meta-narrative".ti,ab,kf,kw OR "Meta-interpretation".ti,ab,kf,kw OR "Meta-Analysis".ti,ab,kf,kw OR "Umbrella review?".ti,ab,kf,kw OR "systematic review?".ti,ab,kf,kw OR "mapping review?".ti,ab,kf,kw OR (Integrative OR Realist) adj2 (Review? OR Synthesis).ti,ab,kf,kw OR ((comprehensive OR literature OR "State-of-the-art") adj1 Review?).ti,ab,kf,kw |  |
| 10 | 8 OR 9 |  |
| 11 | 4 AND 7 |  |
| 12 | (1 OR 11) AND 10 | 245 |

**Embase (Embase.com)**

**Date of the search:** 07-03-2024

**Database limit:** No database limit has been applied

| **#** | **Search strategy** | **Results** |
| --- | --- | --- |
| 1 | teledentistry/de |  |
| 2 | Teledentistry:ti,ab,kw OR "tele-dentistry":ti,ab,kw |  |
| 3 | #1 OR #2 |  |
| 4 | 'dentistry'/exp OR 'mouth tumor'/exp OR Dentists/de OR Dentistry/exp OR 'stomatology'/de OR 'oral surgery'/de |  |
| 5 | dental:ti,ab,kw OR dentist*:ti,ab,kw OR orthodonti*:ti,ab,kw OR periodont*:ti,ab,kw OR prosthodont*:ti,ab,kw OR (oral NEAR/2 (health OR care OR surger* OR Diagnos* OR Hygiene OR Medicine OR lesion$)):ti,ab,kw OR ((oral OR Mouth) NEAR/1 (cancer OR Neoplasm$)):ti,ab,kw |  |
| 6 | #4 OR #5 |  |
| 7 | telemedicine/de OR telediagnosis/de OR "video consultation"/de OR "teleconsultation"/exp OR "mobile application"/exp OR "mobile phone"/exp OR "text messaging"/de OR videoconferencing/de OR "web conferencing"/de |  |
| 8 | Telemedicine:ti,ab,kw OR Telehealth:ti,ab,kw OR Teleconsultation:ti,ab,kw OR telediagnosis:ti,ab,kw OR teletriage:ti,ab,kw OR telemonitoring:ti,ab,kw OR mHealth:ti,ab,kw  OR eHealth:ti,ab,kw OR "e-health":ti,ab,kw OR "Mobile Health":ti,ab,kw OR (remote NEAR/2 (telecommunication OR Consultation OR care OR diagnosis)):ti,ab,kw OR (tele NEAR/2 (medicine OR consultation* OR health OR diagnosis OR triage)):ti,ab,kw OR (virtual NEAR/2 (consult* OR care)):ti,ab,kw OR econsult*:ti,ab,kw OR "e consult*":ti,ab,kw OR ((Video OR Electronic) NEAR/2 Consult*):ti,ab,kw OR "mobile app*":ti,ab,kw OR "cellular phone":ti,ab,kw OR smartphone*:ti,ab,kw OR "text messag*":ti,ab,kw OR "mobile phone":ti,ab,kw OR Videoconferenc*:ti,ab,kw |  |
| 9 | #7 OR #8 |  |
| 10 | 'meta analysis'/exp OR 'review'/de OR 'systematic review'/de |  |
| 11 | (scoping NEAR/2 (stud* OR review$)):ti,ab,kw OR (evidence NEAR/2 map*):ti,ab,kw OR "Environmental scan*":ti,ab,kw OR "evidence synthesis":ti,ab,kw OR "systematic map*":ti,ab,kw  OR overview$:ti,ab,kw OR "review of review$":ti,ab,kw OR "rapid review$":ti,ab,kw OR (Narrative NEAR/1 (review OR summary OR Synthesis)):ti,ab,kw OR (mixed NEAR/1 (method$ OR stud*)):ti,ab,kw OR "Research Synthesis":ti,ab,kw OR "Meta-synthesis":ti,ab,kw OR "Meta-Theory":ti,ab,kw OR "Meta-Study":ti,ab,kw OR "Meta Ethnography":ti,ab,kw OR "Meta-aggregation":ti,ab,kw OR "Meta-narrative":ti,ab,kw OR "Meta-interpretation":ti,ab,kw OR "Meta-Analysis":ti,ab,kw OR "Mapping Review$":ti,ab,kw OR "Umbrella review$":ti,ab,kw OR "systematic review$":ti,ab,kw OR ((Integrative OR Realist) NEAR/2 (Review$ OR Synthesis)):ti,ab,kw OR ((comprehensive OR literature OR "State-of-the-art") NEAR/1 Review$):ti,ab,kw |  |
| 12 | #10 OR #11 |  |
| 13 | #6 AND #9 |  |
| 14 | (#3 OR #13) AND #12 | 392 |

**CINAHL (EBSCO)**

**Date of the search:** 07-03-2024

**Database limit:** No database limit has been applied

| **#** | **Search strategy** | **Results** |
| --- | --- | --- |
| 1 | MH Teledentistry |  |
| 2 | TI Teledentistry OR AB Teledentistry OR TI "tele-dentistry" OR AB "tele-dentistry" |  |
| 3 | S1 OR S2 |  |
| 4 | MH "Dentistry+" OR MH "Mouth Neoplasms" OR MH "Dentists+" OR MH "Surgery, Oral+" OR MH "Specialties, Dental+" |  |
| 5 | TI dental OR AB dental OR TI dentist* OR AB dentist* OR TI orthodonti* OR AB orthodonti*  OR TI periodont* OR AB periodont* OR TI prosthodont* OR AB prosthodont* OR TI (oral N2 (health OR care OR surger* OR Diagnos* OR Hygiene OR Medicine OR lesion#)) OR AB (oral N2 (health OR care OR surger* OR Diagnos* OR Hygiene OR Medicine OR lesion#)) OR TI ((oral OR Mouth) N1 (cancer OR Neoplasm#)) OR AB ((oral OR Mouth) N1 (cancer OR Neoplasm#)) |  |
| 6 | S4 OR S5 |  |
| 7 | MH "Telemedicine+" OR MH "Videoconferencing+" OR MH "Mobile Applications"  OR MH "Cellular Phone" OR MH "Text Messaging" OR MH "Smartphone" |  |
| 8 | TI Telemedicine OR AB Telemedicine OR TI Telehealth OR AB Telehealth OR TI Teleconsultation OR AB Teleconsultation OR TI telediagnosis OR AB telediagnosis OR TI teletriage OR AB teletriage OR TI telemonitoring OR AB telemonitoring OR TI mHealth OR AB mHealth OR TI eHealth OR AB eHealth OR TI "e-health" OR AB "e-health" OR TI "Mobile Health" OR AB "Mobile Health" OR TI (remote N2 (telecommunication OR Consultation OR care OR diagnosis)) OR AB (remote N2 (telecommunication OR Consultation OR care OR diagnosis)) OR TI (tele N2 (medicine OR consultation* OR health OR diagnosis OR triage)) OR AB (tele N2 (medicine OR consultation* OR health OR diagnosis OR triage)) OR TI (virtual N2 (consult* OR care)) OR AB (virtual N2 (consult* OR care)) OR TI econsult* OR AB econsult* OR TI "e consult*" OR AB "e consult*" OR TI ((Video OR Electronic) N2 Consult*) OR AB ((Video OR Electronic) N2 Consult*) OR TI "mobile app*" OR TI "cellular phone" OR TI smartphone* OR TI "text messag*" OR TI "mobile phone" OR AB "mobile app*" OR AB "cellular phone" OR AB smartphone* OR AB "text messag*" OR AB "mobile phone" |  |
| 9 | S7 OR S8 |  |
| 10 | MH "Literature Review+" OR MH "Meta Synthesis" OR MH "Meta Analysis" |  |
| 11 | TI (scoping N2 (stud* OR review#)) OR AB (scoping N2 (stud* OR review#)) OR TI (evidence N2 map*) OR AB (evidence N2 map*) OR TI "Environmental scan*" OR AB "Environmental scan*" OR TI "evidence synthesis" OR AB "evidence synthesis" OR TI "systematic map*" OR AB "systematic map*" OR TI overview# OR AB overview# OR TI "review of review#" OR AB "review of review#" OR TI rapid review? OR AB rapid review? OR TI (Narrative N1 (review OR summary OR Synthesis)) OR AB (Narrative N1 (review OR summary OR Synthesis)) OR TI (mixed NEAR/1 (method? OR stud*)) OR AB (mixed NEAR/1 (method? OR stud*)) OR TI "Research Synthesis" OR AB "Research Synthesis" OR TI "Meta-synthesis" OR AB "Meta-synthesis" OR TI "Meta-Theory" OR AB "Meta-Theory" OR TI "Meta-Study" OR AB "Meta-Study" OR TI "Meta Ethnography" OR AB "Meta Ethnography" OR TI "Meta-aggregation" OR AB "Meta-aggregation" OR TI "Meta-narrative" OR AB "Meta-narrative" OR TI "Meta-interpretation" OR AB "Meta-interpretation" OR TI "Meta-Analysis" OR AB "Meta-Analysis" OR TI "Umbrella review#" OR AB "Umbrella review#" OR TI "systematic review#" OR AB "systematic review#" OR TI "mapping review#" OR AB "mapping review#" OR TI (Integrative OR Realist) N2 (Review# OR Synthesis) OR AB (Integrative OR Realist) N2 (Review# OR Synthesis) OR TI ((comprehensive OR literature OR "State-of-the-art") N1 Review#) OR AB ((comprehensive OR literature OR "State-of-the-art") N1 Review#) |  |
| 12 | S10 OR S11 |  |
| 13 | S6 AND S9 |  |
| 14 | (S3 OR S13) AND S12 | 91 |

**Web of Science**

**Date of the search:** 07-03-2024

**Database limit:** No database limit has been applied

| **#** | **Search strategy** | **Results** |
| --- | --- | --- |
| 1 | TS=Teledentistry OR TS="tele-dentistry" |  |
| 2 | TS=dental OR TS=dentist* OR TS=orthodonti* OR TS=periodont* OR TS=prosthodont* OR TS=(oral NEAR/2 (health OR care OR surger* OR Diagnos* OR Hygiene OR Medicine OR lesion$)) OR TS=((oral OR Mouth) NEAR/1 (cancer OR Neoplasm$)) |  |
| 3 | TS=Telemedicine OR TS=Telehealth OR TS=Teleconsultation OR TS=telediagnosis OR TS=teletriage OR TS=telemonitoring OR TS=mHealth OR TS=eHealth OR TS="e-health" OR TS="Mobile Health" OR TS=(remote NEAR/2 (telecommunication OR Consultation OR care OR diagnosis)) OR TS=(tele NEAR/2 (medicine OR consultation* OR health OR diagnosis OR triage)) OR TS=(virtual NEAR/2 (consult* OR care)) OR TS=econsult* OR TS="e consult*" OR TS=((Video OR Electronic) NEAR/2 Consult*) OR TS="mobile app*" OR TS="cellular phone" OR TS=smartphone* OR TS="text messag*" OR TS="mobile phone" OR TS=Videoconferenc* |  |
| 4 | #2 AND #3 |  |
| 5 | TS=(scoping NEAR/2 (stud* OR review$)) OR TS=(evidence NEAR/2 map*) OR TS="Environmental scan*" OR TS="evidence synthesis" OR TS="systematic map*" OR TS=overview$ OR TS="review of review$" OR TS="rapid review$" OR TS=(Narrative NEAR/1 (review OR summary OR Synthesis)) OR TS=(mixed NEAR/1 (method$ OR stud*)) OR TS="Research Synthesis" OR TS="Meta-synthesis" OR TS="Meta-Theory" OR TS="Meta-Study"  OR TS="Meta Ethnography" OR TS="Meta-aggregation" OR TS="Meta-narrative" OR TS="Meta-interpretation" OR TS="Meta-Analysis" OR TS="Umbrella review$" OR TS="systematic review$" OR TS="mapping review$" OR TS=((Integrative OR Realist) NEAR/2 (Review$ OR Synthesis)) OR TS=((comprehensive OR literature OR "State-of-the-art") NEAR/1 Review$) |  |
| 6 | (#1 OR #4) AND #5 | 223 |

**Cochrane Library**

**Date of the search:** 07-03-2024

**Database limit:** limit results to systematic review only

| **#** | **Search strategy** | **Results** |
| --- | --- | --- |
| 1 | Teledentistry:ti,ab,kw OR "tele-dentistry":ti,ab,kw |  |
| 2 | [mh ^"Dental Health Services"] OR [mh ^Dentists] OR [mh Dentistry] OR [mh "Mouth Neoplasms"] |  |
| 3 | dental:ti,ab,kw OR dentist*:ti,ab,kw OR orthodonti*:ti,ab,kw OR periodont*:ti,ab,kw OR prosthodont*:ti,ab,kw OR (oral NEAR/2 (health OR care OR surger* OR Diagnos* OR Hygiene OR Medicine OR lesion*)):ti,ab,kw OR ((oral OR Mouth) NEAR/1 (cancer OR Neoplasm*)):ti,ab,kw |  |
| 4 | #2 OR #3 |  |
| 5 | [mh Telemedicine] OR [mh ^"Mobile Applications"] OR [mh "Cell Phone"] OR [mh Videoconferencing] |  |
| 6 | Telemedicine:ti,ab,kw OR Telehealth:ti,ab,kw OR Teleconsultation:ti,ab,kw OR telediagnosis:ti,ab,kw OR teletriage:ti,ab,kw OR telemonitoring:ti,ab,kw OR mHealth:ti,ab,kw OR eHealth:ti,ab,kw OR "e-health":ti,ab,kw OR "Mobile Health":ti,ab,kw OR (remote NEAR/2 (telecommunication OR Consultation OR care OR diagnosis)):ti,ab,kw OR (tele NEAR/2 (medicine OR consultation OR health OR diagnosis OR triage)):ti,ab,kw OR (virtual NEAR/2 (Consultation OR care)):ti,ab,kw OR econsultation:ti,ab,kw OR "e consultation":ti,ab,kw OR ((Video OR Electronic) NEAR/2 Consultation):ti,ab,kw OR "mobile app":ti,ab,kw OR "mobile application" OR "cellular phone":ti,ab,kw OR smartphone:ti,ab,kw OR "text messaging":ti,ab,kw OR "mobile phone":ti,ab,kw OR Videoconference:ti,ab,kw OR videconferencing:ti,ab,kw |  |
| 7 | #5 OR #6 |  |
| 8 | #4 AND #7 |  |
| 9 | #8 OR #1 |  |
| 10 | *Database Systematic review limit only* | 19 |

**Epistemonikos (https://www.epistemonikos.org/)**

**Date of the search:** 07-03-2024

**Database limit:** limit results to systematic review only

| **#** | **Search strategy** | **Results** |
| --- | --- | --- |
| 1 | (title:((title:((Teledentistry OR "tele-dentistry") OR ((dentist* OR dental OR "oral health") AND (Telemedicine OR Telehealth OR Teleconsultation OR telediagnosis OR teletriage OR telemonitoring OR "remote consult*"))) OR abstract:((Teledentistry OR "tele-dentistry") OR ((dentist* OR dental OR "oral health") AND (Telemedicine OR Telehealth OR Teleconsultation OR telediagnosis OR teletriage OR telemonitoring OR "remote consult*"))))) OR abstract:((title:((Teledentistry OR "tele-dentistry") OR ((dentist* OR dental OR "oral health") AND (Telemedicine OR Telehealth OR Teleconsultation OR telediagnosis OR teletriage OR telemonitoring OR "remote consult*"))) OR abstract:((Teledentistry OR "tele-dentistry") OR ((dentist* OR dental OR "oral health") AND (Telemedicine OR Telehealth OR Teleconsultation OR telediagnosis OR teletriage OR telemonitoring OR "remote consult*")))))) | 50 |
