# Supplementary material for: Teledentistry for Improving Access To, and Quality of Oral Health Care: Overview of Systematic Reviews and Meta-Analyses
Source: J Med Internet Res. 2025 Jul 30;27:e65211. doi: 10.2196/65211 (PMC12334114; doi:10.2196/65211)
Supplement: Multimedia Appendix 2 [file jmir-v27-e65211-s002.docx]

**Tables of excluded studies with reasons**

| **No.** | **Excluded references** | **Reasons** |
| --- | --- | --- |
| 1. | Abaza H, Marschollek M. mHealth Application Areas and Technology Combinations*. A Comparison of Literature from High and Low/Middle Income Countries. Methods Inf Med. 2017 Aug 8;56(7):e105-e122. | Non SR |
| 2. | Neville P, van der Zande MM. Dentistry, e-health and digitalisation: A critical narrative review of the dental literature on digital technologies with insights from health and technology studies. Community Dent Health. 2020 Feb 27;37(1):51-58. | Non SR |
| 3. | Ai-Shammerya D, Alqhtani N, Alotaibi AN, AlSharidah M, AlShehri K, AlShamrani A. Contributions and Concerns about the Use of Teledentistry in Clinical Orthodontics. Oral Health Prev Dent. 2021 Jan 7;19(1):465-469. | Non SR |
| 4. | Vučinić D, Vukman R, Devčić, MK, Prpić J. Advantages of new technologies during the COVID-19 pandemic. Acta Stomatologica Croatica. 2021;55(2):234 | Non SR |
| 5. | Daniel SJ, Wu L, Kumar S. Teledentistry: a systematic review of clinical outcomes, utilization and costs. J Dent Hyg. 2013 Dec;87(6):345-52. | Other eligibility criteria |
| 6. | Aljohani M, Aldosari B, Alanazi A, Alotaibi G. Effect of Teledentistry and Outcome for Dental Professionals at Saudi Arabia: A Systematic scoping Review. J Res Med Dent Sci. 2021;9(8): 157-162. | Other eligibility criteria |
| 7. | Squires T, Michelogiannakis D, Rossouw PE, Javed F. An evidence-based review of the scope and potential ethical concerns of teleorthodontics. J Dent Educ. 2021 Jan;85 (1):92-100. | Other eligibility criteria |
| 8. | Besimo CE, Zitzmann NU, Joda T. Digital Oral Medicine for the Elderly. Int. J. Environ. Res. Public Health. 2020 Mar 25;17(7):2171. | Non SR |
| 9. | Adebayo B, Durey A, Slack-Smith LM. Role of information and communication technology in promoting oral health at residential aged care facilities. Aust J Prim Health. 2017 Jul;23(3):216-222. | Non SR |
| 10. | Achmad H, Tanumihardja, M, Ramadhany YF. Teledentistry as a solution in dentistry during the covid-19 pandemic period: A systematic review. International Journal of Pharmaceutical Research. 2020;12():272-278 | Other eligibility criteria |
| 11. | Matsuda S, Yoshimura H. Possibilities and challenges in digital personal identification using teledentistry based on integration of telecommunication and dental information: a narrative review. J Int Med Res. 2022 Apr;50(4):3000605221097370. | Non SR |
| **No.** | **Excluded references** | **Reasons** |
| 12. | Mariño R, Ghanim A. Teledentistry: a systematic review of the literature. J Telemed Telecare. 2013 Jun;19(4):179-83. | Other eligibility criteria |
| 13. | Discepoli N, Mirra R, Marruganti C, Beneforti C, Doldo T. Efficacy of Behaviour Change Techniques to improve oral hygiene control of individuals undergoing orthodontic therapy. A systematic review. Int J Dent Hyg. 2021 Feb;19(1):3-17. | Not teledentistry |
| 14. | Teixeira CNG, Rodrigues, MIQ, Frota, LMA, Frota MMA, Oliveira AEF. Situational panorama of Teleodontology in the world: an integrative review. Rev. ABENO. 2018;18(3):24-34 | Not English or French language |
| 15. | Cunningham, A., McPolin, O., Fallis, R. et al. A systematic review of the use of virtual reality or dental smartphone applications as interventions for management of paediatric dental anxiety. BMC Oral Health. 2021;21(1):244 | Other eligibility criteria |
| 16. | Chen R, Santo K, Wong G, Sohn W, Spallek H, Chow C, Irving M Mobile Apps for Dental Caries Prevention: Systematic Search and Quality Evaluation. JMIR Mhealth Uhealth. 2021 Jan 13;9(1):e19958. | Non SR |
| 17. | Jokstad A. Computer-assisted technologies used in oral rehabilitation and the clinical documentation of alleged advantages - a systematic review. J Oral Rehabil. 2017 Apr;44(4):261-290. | Not teledentistry |
| 18. | Garfan S, Alamoodi AH, Zaidan BB, Al-Zobbi M, Hamid RA, Alwan JK, Ahmaro IYY, Khalid ET, Jumaah FM, Albahri OS, Zaidan AA, Albahri AS, Al-Qaysi ZT, Ahmed MA, Shuwandy ML, Salih MM, Zughoul O, Mohammed KI, Momani F. Telehealth utilization during the Covid-19 pandemic: A systematic review. Comput Biol Med. 2021 Nov;138:104878. | Non dental setting |
| 19. | Mir C. Among orthodontic patients, does the use of reminder systems compared to no reminders improve periodontal parameters and appointment adherence? Evid Based Dent. 2019 Sep;20(3):94. | Non SR |
| 20. | Chandran VP, Balakrishnan A, Rashid M, Pai Kulyadi G, Khan S, Devi ES, Nair S, Thunga G. Mobile applications in medical education: A systematic review and meta-analysis. PLoS One. 2022 Mar 24;17(3):e0265927. | Non dental setting |
| 21. | da Silva HEC, Sagntos GNM, Ferreira Leite A, Mesquita CRM, de Souza Figueiredo PT, Miron Stefani C, de Santos Melo N. The feasibility of telehealth in the monitoring of head and neck cancer patients: a systematic review on remote technology, user adherence, user satisfaction, and quality of life. Support Care Cancer. 2022 May 6. | Non dental setting |
| 22. | Car J, Gurol-Urganci I, de Jongh T, Vodopivec-Jamsek V, Atun R, Car J. Mobile phone messaging reminders for attendance at healthcare appointments. Cochrane Database Syst Rev. 2013 Dec 5;2013(12):CD007458. | Non dental setting |
| **No.** | **Excluded references** | **Reasons** |
| 23. | Badawy SM, Kuhns LM. Texting and Mobile Phone App Interventions for Improving Adherence to Preventive Behavior in Adolescents: A Systematic Review. JMIR Mhealth Uhealth. 2017 Apr 19;5(4):e50. | Non dental setting |
| 24. | Sharma S, Mohanty V, Balappanavar A Y, et al. (September 07, 2022) Role of Digital Media in Promoting Oral Health: A Systematic Review. Cureus 14(9): e28893. DOI 10.7759/cureus.28893 | Other eligibility criteria |
| 25. | Inês Meurer M, Caffery LJ, Bradford NK, Smith AC. Accuracy of dental images for the diagnosis of dental caries and enamel defects in children and adolescents: A systematic review. *J Telemed Telecare*. 2015;21(8):449-458. | Other eligibility criteria |
| 26. | Chatterjee S, Khan AM, Rani P, Jayesh, Shankar D. Systematic review on tele dentistry in public oral health during covid- 19. Neuro Quantology. 2022;20 (7):1107-1117 | Other eligibility criteria |
| 27. | Kui A, Popescu C, Labuneț A, et al. Is Teledentistry a Method for Optimizing Dental Practice, Even in the Post-Pandemic Period? An Integrative Review. *Int J Environ Res Public Health*. 2022;19(13):7609. | Non SR |
| 28. | Gurgel-Juarez N, Torres-Pereira C, Haddad AE, et al. Accuracy and effectiveness of teledentistry: a systematic review of systematic reviews [published online ahead of print, 2022 Jul 8]. *Evid Based Dent*. 2022;1-8. | Non SR |
| 29. | Wafaie K, Rizk MZ, Basyouni ME, Daniel B, Mohammed H. Tele-orthodontics and sensor-based technologies: a systematic review of interventions that monitor and improve compliance of orthodontic patients. *Eur J Orthod*. 2023;45(4):450-461. | Non SR |
| 30. | Mohammadzadeh N, Gholamzadeh M, Zahednamazi S, Ayyoubzadeh SM. Mobile health applications for children's oral health improvement: A systematic review. *Informatics in*  *Medicine Unlocked*, 2023.101189. | Other eligibility criteria |
| 31. | Sarbaz M, Monazah FM, Eslami S, Kimiafar K, Baigi SFM. Effect of mobile health interventions for side effects management in patients undergoing chemotherapy: A systematic review. [*Health Policy and Technology*](https://www.sciencedirect.com/journal/health-policy-and-technology). 2022;11(4):100680 | Non dental setting |
| 32. | Alam MK, Abutayyem H, Kanwal B, Shayeb M. Future of Orthodontics-A Systematic Review and Meta-Analysis on the Emerging Trends in This Field. *J Clin Med*. 2023;12(2):532 | Wrong intervention |
| **No.** | **Excluded references** | **Reasons** |
| 33. | ElNaghy R, Al-Qawasmi R, Hasanin M. Does using mobile applications and social media-based interventions induce beneficial behavioral changes among orthodontic patients? *Evid Based Dent*. 2023;24(1):26-27. | Commentary |
| 34. | Patil S, Hedad IA, Jafer AA, et al. Effectiveness of mobile phone applications in improving oral hygiene care and outcomes in orthodontic patients. *J Oral Biol Craniofac Res*. 2021;11(1):26-32 | Wrong eligibility criteria |
| 35. | Kaneyasu Y, Shigeishi H, Sugiyama M, Ohta K. Effectiveness of e-learning to promote oral health education: A systematic review and meta-analysis. *Medicine* (Baltimore). 2023 Dec 22;102(51):e36550. | Wrong intervention |
| 36. | Maita KC, Palmieri-Serrano L, Avila FR. *et al.* Imaging evaluated remotely through telemedicine as a reliable alternative for accurate diagnosis: a systematic review. *Health Technol.* 2023;13, 347–364. | Wrong setting |
| 37. | de Oliveira Júnior AJ, Oliveira JM, Bretz YP, Mialhe FL. Online social networks for prevention and promotion of oral health: a systematic review. *Can J Dent Hyg.* 2023 Jun 1;57(2):83-97. | Wrong intervention |
| 38. | Sharma D, Gupta S, Koshy G, Sharma VK, Hooda A. Potential implications of mobile applications in oral cancer. *J Cancer Res Ther.* 2023 Oct 1;19(7):1691-1696. | Wrong study design |
| 39. | Silva VAN, Cunha R, Leite ICG. COVID-19 Pandemic and applicability of teledentistry in primary health care based on international experiences. *Rev. Ciênc. Plur* 2022;8(2):e26130-e26130. | Wrong study design |
