# Supplementary material for: Teledentistry for Improving Access To, and Quality of Oral Health Care: Overview of Systematic Reviews and Meta-Analyses
Source: J Med Internet Res. 2025 Jul 30;27:e65211. doi: 10.2196/65211 (PMC12334114; doi:10.2196/65211)
Supplement: Multimedia Appendix 3 [file jmir-v27-e65211-s003.docx]

**Characteristics of included studies**

| **Review author, year** | **No. of included studies (sample size), study designs** | **Population** | **Settings** | **Domains in dentistry** | **Intervention (modality)** | **Control** | **Outcomes** | **Main conclusions** | **Review limitations** |
| --- | --- | --- | --- | --- | --- | --- | --- | --- | --- |
| Abdul et al., 2023 ^1^ | 2 (753)  RCT, quasi experimental study | Patients irrespective of age, gender and ethnicity | NR | Oral medicine | Telemedicine  (Synchronous and asynchronous) | Conventional treatment or diagnosis | Diagnosis, functional outcome of pain and  quality of life such as loss of working hours | T-Med is a promising modality for TMD management. T-Med can improve access to care, reduce the burden on healthcare facilities, and provide a more comfortable and convenient experience for patients. T-Med can also provide patients  with greater control over their care by enabling them to manage their symptoms and communicate with healthcare professionals  more easily. | Only a few relevant studies identified, which could  restrict the generalizability and robustness of the findings. The included studies utilized different telemedicine modalities that could have probably affected the overall consistency and effectiveness of telemedicine in TMD diagnosis and management. |
| Alabdullah & Daniel, 2018 ^2^ | 9 (1035)  Cross-sectional studies, RCT, Cohort longitudinal study RCT | Children and young adults  from 1 year to 19 years of age, and adults (>20 years) | Clinical setting and in a laboratory | Pediatric dentistry, general dentistry, maxillofacial radiology, endodontics, and orthodontics | An intraoral camera to gather images of teeth for later examination, a digital extraoral  camera and a smartphone camera. Wireless telemedicine system, remote computer, smartphone camera, cloud storage, digital and intraoral camera, file sharing service, store and forward system, digital camera, e-mail.  (Asynchronous) | Gold standard control reported was the traditional visual examination. Methods and items used for visual examinations included the following: use of light  source, sterilized exploration kit, probe, air syringe, mirror, explorer, and palpation. | Validity of teledentistry including visual clinical examination for dental caries assessment | Teledentistry could be a comparable tool to face-to-face technology for  oral screening especially for school-based programs, caries assessment, referrals, and teleconsultations. teledentistry is a viable technology for early diagnosis of dental caries among children. Teledentistry is a valid tool to reduce inappropriate orthodontic referrals. | Few studies use research methods and statistical analyses that can provide strong evidence for comparison of traditional visual examination to teledentistry examination. The searched databases and search terms might not identify all the studies published  on teledentistry. Due to methodology variability among studies, it was difficult to generalize findings of this systematic review |
| Al-Moghrabi et al., 2021* ^3^ | 16 (3664)  RCT, non-randomized controlled trials | Orthodontic patients | Dental hospital, university, orthodontic clinics, | Orthodontics | Mobile app-based videos, reminders, written and verbal information, bespoke mobile applications, electric toothbrush with a mobile application connected to *Bluetooth*, *Instagram* posts, social medias  Apps: Youtube, *Whatsapp*, *White teeth* mobile app  (Asynchronous) | Manual toothbrush, verbal and written information | Primary: adherence to wearing of appliances or adjuncts, oral health-related behaviors, oral hygiene levels,  Periodontal outcomes, and related iatrogenic effects, white spot  lesions, and caries, appointment attendance, knowledge  Secondary: patient-reported outcomes and treatment-related experiences | The intervention was favored in relation to gingival and plaque indexes, standardized mean difference: -0.81 (95% confidence intervals [CI], -1.35 to -0.28) and -0.91 (95% CI, -1.64 to -0.19), respectively. However, no significant effect was observed in bleeding on probing (standardized mean difference: -0.22; 95% CI, -0.5 to 0.05). A very low to moderate level of evidence supporting the effects of mobile applications and social media-based interventions in producing positive behavioral changes in orthodontic patients.  Weak evidence to support the effectiveness of mobile applications and social media-based interventions on plaque and gingival scores among orthodontic patients. | Very low and moderate level of evidence for most primary outcome measures. The settings limit the generalizability of results. The follow-up period was relatively short. Sustainability of behavioral change could not be ascertained. Publication bias was not assessed because of an insufficient number of studies included in meta-analysis. |
| Aquilanti et al., 2020 ^4^ | 6 (778)  Feasibility study and cost-analysis, cross-sectional and mixed methods studies, reports | Elders aged 65 years and over | Residential aged care facilities, nursing homes, long term facilities and specialized facilities for adults with severe intellectual, motor or somatic disabilities | Public health | Teledentistry for Screening, diagnosis, support, consultation, education, and any other kind  of application in dental medicine.  (Synchronous and asynchronous) | Traditional face-to-face dental visits | Primary: accuracy and effectiveness  of teledentistry. Minimization of need to attend an oral health care facility; Onset of dispositional resistance; Onset of anxiety regarding the procedure  Secondary: assessment of  advantages and disadvantages of teledentistry.  Evaluation of patient acceptability and cost effectiveness. | The implementation of teledentistry in residential aged care facilities and in home-assistance programs could be a viable tool for the management of oral care in people who cannot access dental care.  There is a strong trend supporting the feasibility of teledentistry compared to the traditional approaches. However, conclusive statements cannot  be drawn. | Absence of high-quality studies limited the findings. A high heterogeneity was present among the articles reporting different outcomes. Possibility of publication bias. |
| Bhamra et al., 2024^5^ | 4 (684)  Cross- sectional studies | Care home residents | residential aged-care  facilities, nursing homes | NR | Teledentistry  (Synchronous and asynchronous) | In person examination, face to face consultations | Feasibility, experiences, impact, access to care, acceptability, efficacy and  Economics. | Teleconsultations  were shown to have great potential to care planning/follow-up rather than physical examinations or assessments with  some evidence of diagnostic accuracy. | Only records written in English were included, which  limited the search.  Heterogeneity of the studies included in terms of location,  and analysis conducted. None of the studies  considered the underlying digital literacy amongst the social  care workforce, or patient population,  the regulatory aspects, equipment or infrastructure challenges  of a digital landscape. |
| BöhmdaCosta et al., 2019 ^6^ | 24  Cross-sectional, mixed-methods studies, case reports | Dental patients, including pediatric, orthodontic, and elderly patients, prisoners | NA | Dental public health | Teledentistry  (Synchronous and asynchronous) | NA | Issues faced in relation to the use of teledentistry, its advantages, and the obstacles encountered in its  application | Teledentistry can be used for training and continuing education of professionals, for remote patient care, to exchange information among health professionals. Benefits include reduction of waiting time, avoiding expenses related to displacements, saving financial resources, and increased access to dental and specialist care in remote areas. It enables interactions between general practitioners with colleagues from different dental specialties, thereby increasing the quality of the care provided  and improving patient satisfaction. | Search strategy, only targeted teledentistry applications in public dental health services in a community context, over a restrictive time period. Only articles published in one language was included in the review. Limited evidence to formulate  definitive conclusions on the best type of teledentistry for public health needs. |
| Chau et al.,2023^7^ | 5 (422)  RCT, quasi experimental study | Elders (>60years) | Social service program, community, dental clinic | NR | Oral health education using teledentistry.  (Asynchronous) | No oral health education, oral health education leaflets | Oral health knowledge, behavior, OHIP-14, clinical outcomes | mHealth is being used by dental professionals  to improve oral health management, oral health behavior, and oral health knowledge  among older adults with high acceptability and mixed effectiveness. Such technology  may potentially become a valuable tool for promoting oral health. | Most studies did not include baseline clinical examinations, and there was a lack of long-term follow-up. Lack of information about participants’ adherence to the full intervention and the recruitment rate, high dropout rates.  No meta-analysis, limited generalizability. |
| Choi et al., 2021^8^ | 11 (1164)  RCT | Adolescents, adults, | Clinical setting | Orthodontics | Mail, Telephone, Text messages, WeChat App  (Asynchronous) | Control group did not receive notifications. Patients not receiving the text messages, reminders, conventional management, oral hygiene instruction | Primary: plaque índices, periodontales índices,  proportion or number of white-spot lesion  Secondary: percentage of patient attendance, levels of overall self-reported pain and sagittal distance. | Mobile health care can be utilized as an adjuvant intervention to improve treatment outcomes in patients undergoing fixed orthodontic  treatment. To improve the oral health of patients undergoing fixed  orthodontic treatment, oral healthcare experts must promote the  active participation of patients using not only conventional interventions, but also mobile health care. | Some selected studies were of poor quality with potential bias, resulting in a risk of bias in the entire review. In addition,  most interventions in the included studies lasted 12 weeks or less,  which may be insufficient to identify the long-term effects of using  a mobile device-based healthcare intervention. Furthermore, different indices were used among the selected studies, and therefore,  qualitative synthesis was performed because quantitative synthesis was not possible |
| da Silva et al., 2021^9^ | 11  Letters to the editor/  editorials, case reports | Oral cancer (head and neck) patients,  Oral medicine specialists | NR | NR | Phone, video conference,  or messaging through applications.  Apps: *Zoom* app  TeleHealth system,  *WhatsApp*  (Synchronous and asynchronous) | NR | Performance, management, control, and assistance through  teledentistry delivered by dental surgeons (specialized in oral and maxillofacial surgery or members of a multidisciplinary  oncology treatment team) to patients with oral and head and neck cancer during the COVID-19 pandemic. | Included studies showed that teledentistry applied to monitoring of patients with oral cancer and head and neck cancer  was a useful tool in supporting patients during the pandemic, improved general well-being and quality of life.  Teledentistry was well accepted as a remote technology tool for monitoring oral and head and neck cancer patients. It was well accepted by patients and professionals in preliminary studies.  It guaranteed access to technology by  low-income people, optimised care, reduced waiting time for an appointment, and increased the resolution of the clinical cases. | Few studies in literature correlating  teledentistry and the monitoring of patients with oral and head and neck cancer, both in the direct care of dental complications during cancer treatment and by aggravation caused by  the cancer treatment itself. |
| deLima et al., 2024*^10^ | 6 (2064)  Observational studies | Patients with and without pre-malignant oral lesions | NR | NR | mHealth  (Asynchronous) | Clinical examination of oral cancer | Accuracy, sensitivity, specificity, predictive value | A high probability of  detection of PMOL by remote examination (97.37%) was  observed for the three studies included in the meta-analysis,  with high heterogeneity among them. The certainty of evidence for the outcome was considered very low  The results of this systematic review point to a high probability of diagnostic accuracy by remote examination for  detecting PMOLs, and these technologies are feasible for use in  regions with difficult access for specialists. | Limitations related to mall number of studies and methodological quality. |
| Emami et al., 2022^11^ | 16 (6378+2.7 million children-Australian health survey))  Cross-sectional study, Non-randomized trial, Observational study, Quasi-experimental, Cost-analysis, mixed method study | Patients in rural and remote areas | Schools, general practice, central hospital, regional health centres, university hospital, rural dental clinic, primary care hospitals, residential aged care facilities, | Oral and maxillofacial surgery, oral pathology, oral medicine, prosthodontics | Teledentistry consultations, either live or store and forward, smartphone-based applications like WhatsApp, Oncogrid application for oral cancer surveillance, videographic examination.  (Synchronous and asynchronous) | Comparison facilities with and without integrated oral health program, traditional screening methods | Reliability measures for the use of a photo messaging, GOHAI score, Number of appointments avoided, compliance, costs, DMFT. | Patient satisfaction could be associated with several modalities of e-oral health care. E-oral health seems a feasible option for providers who want to contribute to oral care services in rural and remote areas. However, only speculative conclusions can be drawn based on the quality of the included studies. | Narrow inclusion criteria in regard to language and inclusion of various study designs. Most of the included studies had moderate or low quality as well as moderate to critical risk of bias. Furthermore, the reporting of outcomes in the included studies varied considerably. |
| Estai et al., 2016^12^ | 10 (976)  Cross-sectional studies | Children, adolescents and adults, juvenile inmates | Kindergarten, University Oral Health Unit, Primary School, Dental Practice, Hospital, Oral care centre, child care center, juvenile detention facility (private and public) | General and pediatric dentistry | Teledentistry for detection of dental caries.  (Asynchronous) | Traditional face to face examination | Diagnostic performance in the detection of dental caries with teledentistry (specificity and sensitivity) | Teledentistry approach has an acceptable diagnostic value in the detection of caries lesions. Despite very limited published evidence on the diagnostic accuracy of teledentistry, the reviewed studies showed at least comparable results between photographic methods and the non-telemedicine alternatives of caries assessment. The sensitivity of the photographic assessments ranged from 43% to 100% and the specificity ranged from 52% to 100%. Three studies7,17,18 favored the photographic caries assessment to visual examination, and in the remaining 7 studies, the diagnostic accuracy for the photographic method and visual caries assessment were comparable. | Due to the diversity in the research methodology used, the generalization of results may be difficult. All the  selected studies were dominated by uncontrolled  descriptive studies and assessed the efficacy of  teledentistry rather than its effectiveness, thus providing  poor evidence. |
| Estai et al., 2018^13^ | 11(4478)  RCT, non-randomized  controlled study,  Cost-minimisation  study | Children from 8 to11 years, age form 12-60 months, aged care residents, adult patients, adult workers, patients from 38-41 years, age below 4 years, and kids from 2 months -12 years | Urban or rural settings such as  hospitals, clinics, childcare centres or workplaces | Orthodontics, oral medicine, paediatric dentistry, periodontics, prosthodontics, Restorative dentistry, other dental fields | Teleconsultation, telediagnosis, telereferral  (Synchronous and asynchronous) | NR | Clinical outcomes (reliability, effectiveness, efficacy, appropriateness) and cost of teledentistry. | The present review identifies a growing body of evidence  supporting the efficacy of teledentistry, particularly, in  some areas of dentistry. There is a consistent trend in the literature supporting the  validity and reliability of teledentistry applications in comparison to non-telemedicine alternative. There is emerging evidence supporting the efficacy of teledentistry. However, there is not enough conclusive evidence,  particularly for its effectiveness, cost-effectiveness and long-term use, to make evidence-based policy decisions on teledentistry. | Studies provided only preliminary results and considered only the feasibility and short-term use of teledentistry. Due to limited conclusive evidence and the  heterogeneity of the methods used, interventions and outcomes assessed in the reviewed studies, the generalizability  of the findings is limited. There were several limitations in most of the reviewed  studies, so that even though the present review was restricted to good quality publications on teledentistry,  at present they provide an inadequate indication of the  status of this technology. It is possible that a large body  of literature on teledentistry assessment was not located.  Since the focus of this review was only on identifying  controlled assessments of teledentistry and comparative  outcomes, there was no attempt to review all the relevant  grey literature |
| Fernandez eta l., 2021* ^14^ | 19 (2410)  RCT, quasi randomized study | Teenagers and young adults,  with fixed  orthodontics, pediatric patients, adults with mild-moderate  periodontitis | Private clinics, university dental clinics, pediatric  dentistry practice | Orthodontics, pediatrics, general dentistry, periodontics | Text messages and apps combined with educational messages, reinforcement messages, reminders, push notifications and videos. Selfies to evaluate plaque control. Gamification  activities, PowerPoint  slides  Apps: WeChat app,  Telegram channel  Specific app that received gravitational movements from a toothbrush via Bluetooth  telephone calls  (Synchronous and asynchronous) | Traditional healthcare as the standard of practice | Plaque index, gingival index, white spot lesions  Papillary bleeding index, plaque control, patient satisfaction, duration of treatment, knowledge score, behavior, HAPA , oral hygiene index, duration, self-reported frequency of toothbrushing, percentage of bleeding, Correlation between parents’ educational attainment and oral hygiene maintenance of children, community periodontal index | Participants exposed to teledentistry strategies  as opposed to conventional in-person education experienced a reduction in the PI (standardized mean difference, −1.18; 95% CI: −1.54 to −0.82; P < 0.00001, I^2^ = 92%; the GI (standardized mean difference, −2.17; 95% CI, −3.15 to −1.19; P < 0.0001, I^2^ = 97%, and the incidence of WSL  (risk ratio, 0.48; 95% CI, 0.35 to 0.66; P <  0.00001, I^2^ = 0%.  All these differences were statistically significant. In addition, when exploring the effect of teledentistry strategies in the PI  and the GI at 1, 3,  and between 6 and 12months, there was a sustained effect that seemed to increase over time. | Unable to find an explanation for the observed heterogeneity  for outcomes other than gingival index.  Intervention effects across the entire life span could not be obtained.  Outcomes related to behavior modification  were limited. |
| Flores et al., 2020^15^ | 11 (740)  Cross-sectional studies, clinical trials | Doctors, nurses, and nutritionists to dental students, general practitioners, and specialists | Rural areas or remote locations | NR | Clinical photographs and image examination photographs taken  with a smartphone, videoconference, email, questionnaires, histopathological exams, telemedicine applications and platforms  (Synchronous and asynchronous) | NR | Access to dental care for the rural population, reducing travel time for specialized consultations | Teledentistry can assist patients who need specialized diagnosis in  dentistry and specialist consultations.  Good acceptance by patients and professionals. | Heterogeneity of the methodologies used did not allow a meta-analysis. |
| Fortish-Mesa & Hoyos, 2020 ^16^ | 24  Quasi-experimental and cross-sectional studies | Adults, dental professionals and students, children, prisoners, nurses, dieticians | University, urban, rural areas, prison, health centre, school | NR | Applications and mobile devices: iTeethey™, WhatsApp, Remote-I, mobile microscope, digital fluorescence (QLF-D), Soprocare photo camera®.  (Synchronous and asynchronous) | NR | Knowledge and beliefs, DMFT, reliability of  an intraoral camera, videography, sensitivity and specificity, users’  acceptance | This review helped determine the impact of teledentistry in different dental areas. TD may be a viable alternative in the reduction of time, transfers, and costs. | Short-term use of  teledentistry and low level of evidence. Most studies conducted in urban areas and in developed countries. |
| Irving et al., 2018^17^ | 39 (4290)  Observational studies | Children, adults, hospital/emergency patients, dental patients, juvenile detention patients | NR | General dental, oral surgery, orthognathic examination, orthodontics, endodontics,  pediatrics, cleft palate, oral  rehabilitation | Captured images, biopsy results, tooth sectioning to determine depth of demineralization.  (Synchronous and asynchronous) | In person examination, face-to-face supervision, canal positions verified on histological slides | Accuracy or efficiency of teledentistry to assist in analysis, satisfaction rates for participants involved, time and travel advantages  effectiveness of teledentistry to help practitioners with referrals, pediatric consultation, treatment planning, diagnosis and improving periodontal health. | Teledentistry provides a viable option for remote screening, diagnosis, consultation, treatment planning and  mentoring in dentistry with improved cost effectiveness, accuracy and efficient remote assistance for clinicians. | Fair quality of studies.  Publication bias.  Majority of the studies were reported by the developers of the programs, which may have lead to  reporting bias.  Due to different outcome measures, no meta-analysis was performed. |
| Lima et al., 2018*^18^ | 7 (574)  RCT | Pre-adolescent and Adolescents | NR | Orthodontics | Text messaging with or without videos, voice messages, phone calls.  (Asynchronous) | NR | Plaque index, gingival index  and white spots lesions | Reminder therapy showed improved scores for the plaque index  (standardized mean difference=1.22; 95% confidence interval=2.03 to0.42; P=.003) and the  gingival index (standardized mean difference =1.49; 95% confidence interval =2.61 to 0.37; P =  .009). Moreover, there was lower occurrence of white spots (relative risk = 0.53; 95% confidence  interval = 0.38 to 0.74; P = .001) when reminder therapy was implemented.  Reminder therapy may contribute to improvements in plaque and gingival indices and white spots. | Only two articles  were considered for meta-analysis. |
| Lin et al., 2022*^19^ | 6 (6904)  Cross-sectional | Dental educators, dental specialists, general dentists, postgraduate dental students | NR | NR | NA | NA | Knowledge, awareness, attitudes, practices | Based on the single-arm meta-analysis, a  high level of awareness (70.4%, CI: (64.3, 76.5)) and attitude (72.5%, CI: (60.7, 84.3)) towards teledentistry was noted among dental practitioners during the COVID-19 pandemic.  However, the knowledge level (57.9%, CI: (46.0, 69.9)) were deemed moderate, while their  practice level of teledentistry (35.8%, CI: (14.8, 56.8)) was found to be poor among dental  practitioners. | Small number of studies, variation in sample size, different forms of questionnaires were used.  Sampling and response bias of each primary study was not addressed in the review. |
| Mohammed et al., 2019*^20^ | 9 (2078)  RCT | Age range of  12–22 years | Primary, secondary, or a tertiary orthodontic setting. (college, hospital and University) | Orthodontics | mHealth (Active reminders in any form or media aimed at changing oral health behaviour, including reinforcement techniques such as text messages, mobile phone applications, automated systems, emails, phone calls, postal letters).  (Asynchronous) | Only verbal oral hygiene instructions | Primary outcomes:  Periodontal parameters as measured by plaque index, gingival index, and bleeding index,  Rate of attendance  Secondary outcomes:  Total duration of orthodontic treatment, WSLs, dental caries indices, bracket failure rates, patient-reported outcomes, other adverse effects | A modest but statistically significant SMD favoring plaque control in the intervention  group (reminders) was noted (−0.38; 95% CI: −0.65 to −0.10;  P = 0.008; n = 276; I^2^ = 24%). Short-term gingival condition was healthier in  the reminders group (−0.66; 95% CI: −0.97 to −0.35; P < 0.0001;I^2^ = 0%). (11). BI was better in patients receiving reminders; however, results were not statistically significant in the short term (P > 0.05). The long-term (>3 months) PI scores showed a statistically significant SMD favoring reminders (−1.51; 95% CI: −2.72 to −0.30; P = 0.01; n = 394; I2= 95%; 4 trials; Figure 4). The long-term gingival scores showed SMD was statistically significant favoring reminders (−1.94; 95% CI: −3.81 to −0.07; P = 0.04; n = 344; I^2^= 97%). BI was better in patients receiving reminders (P < 0.001). The RR of developing WSLs in the reminders groups was 0.45 (95% CI: 0.31 to 0.65; P < 0.0001; n = 190; I2= 0%; 3 trials). Participants receiving reminders were less likely to develop WSLs during orthodontic treatment. The pooled effects of four RCTs presenting with unclear concerns illustrated that participants were statistically less  likely to fail or cancel their appointments when reminders were sent to them, RR of 0.39 (95% CI: 0.22 to 0.70; P = 0.002;  I^2^= 90%).  Participants in the reminder arm of the study had on  average shorter overall treatment duration compared with control,  MD of 7.3 weeks (95% CI: 3.7 to 11.6).  One RCT  found that participants receiving WeChat application reminders  experienced less bracket failure rates compared with those receiving  no reminders (11.8% versus 16.1 %; P < 0.001). | Many  crucial domains within the included trials were unclear and only two  aggregated studies followed participants for long periods  (12 months or longer) which could present as a limitation when  interpreting the long-term effects. Another limitation was the variable heterogeneity identified across the performed meta-analyses.  The small number of statistically pooled trials hindered the comprehensive interpretation of modifying variables in the treatment. Outcome assessment for periodontal parameters, for instance, could be  easily influenced by operators (ascertainment bias) in their judgement. It was unfortunate that in many included trials, it was unclear  how they handled masking such assessment. |
| Priyank et al., 2023*^21^ | 8 (713),  NR | Children and adults (<four years old to 65 years and older) | NR | NR | Intraoral photographs (using a smartphone), Videographic recording.  (Asynchronous) | NR | Decayed, Missing, Filled Surfaces, Decayed, Missing, Filled Teeth, Dental Caries Severity Scale scores (primary and permanent dentition) | Among the four studies analysed, no significant difference was noted at p = 0.09. A mean difference of 0.64 (95% CI: -0.10; 1.38) suggested that clinical examination and teledentistry-based checkup were on par with each other for the detection of dental caries. Teledentistry and clinical examinations show comparable precision in detecting caries, suggesting that teledentistry can be relied upon as a reliable alternative for caries diagnosis. Teledentistry can serve as a viable alternative for caries screening and detection, potentially supplementing and improving patient care by reducing the need for in-person visits and facilitating remote evaluations. Moreover, teledentistry can be successfully integrated into dental practices with appropriate clinician training and support. | Small sample sizes limit the generalizability of the findings. The age ranges varied across the studies, making it difficult to draw conclusive conclusions for specific age groups. Lack of standardised assessment methods and follow-up periods across the studies. Unspecified follow-up periods made it challenging to compare the efficacy of teledentistry over consistent time frames. No detailed information on potential confounding variables, such as the socioeconomic status or oral health status of the participants. These factors may influence the accuracy of teledentistry in caries detection. |
| Rouanet et al. 2022^22^ | 22 (2103)  Cross-sectional study | Children, teenagers, adults | NR | Orthodontics | Teleorthodontics (Photo, video, WhatsApp, dental monitoring, WeChat, smile consults (Invisalign).  (Synchronous and asynchronous) | NR | Relevance of tools, patient and clinician satisfaction | Teleorthodontics is an interesting and complementary tool that is, in no way, a systematic alternative to face-to-face orthodontic appointments in the office. However, many essential aspects of telemedicine in orthodontics, such as data protection, still need to be investigated in order to fully analyze this tool. | Low certainty of evidence, lack of consideration regarding details such as confidentiality, privacy, security, patient record management. |
| Saccomanno et al., 2022^23^ | 8  NR | Patients facing orthodontic emergencies | Dental clinics | Orthodontics | Video calls (Zoom Video Communications, Inc.), application (Smile Consult by Align Technology Inc., San Jose, CA, USA), Instant messaging, Whatsapp,  Phone calls, Video calls  (Synchronous and asynchronous) | NR | Fracture or loss of brackets, injury to lips or cheek, bracket, tube, band or retainer detachment, gingival inflammation, orthodontic aligner deformation, loss of ligatures, discomfort and pain, oral hygiene. | Teleorthodontics represents a valuable aid for professionals and patients facing orthodontic emergencies. Some of these can be resolved remotely, while others require an emergency visit. With a pre-triage, patients are categorized into one of two options, and emergencies that do not require a visit can be resolved by providing the appropriate indications, depending on the type of equipment and emergency. | Small number of articles,  non homogenous studies |
| Sangalli et al., 2023^24^ | 11 (542)  Case reports, prospective, retrospective studies | Orthodontic patients | NR | Orthodontics | Dental monitoring: a mobile app downloaded on the smartphone of the patient, a web based Doctor Dashboard® managed by the provider, and a movement-tracking algorithm  (GoLive®, 3D Monitoring Light®.  (Asynchronous) | Standard care group without the use of DM | Early detection of orthodontic movement,  patient’s compliance and fit of the aligners, treatment duration, no. of appointments and refinements, aligner tracking, assessing the posterior crossbite, patients’ perspective on the ease of use and benefit to treatment, Plaque Index, gingival Index, white spot lesions, onset of carious lesions, no. of emergency appointments | DM implemented to the standard orthodontic care can significantly decrease the number of in-office visits during an  ongoing orthodontic therapy. The use of DM showed also  some support of improved aligner fit. No difference with treatment duration and number of emergency appointments with DM. | Limited and heterogeneous studies with low methodological rigor, search limited to the English language. |
| Sharif et al., 2019^25^ | 2 (130)  RCT | Children,  Adolescents  (10-18 years) | Community and dental school | Orthodontics | Oral hygiene instructions and dental aids with smartphone-specific video tutorials, and access to a chat room (brush game)  automated text messages  (Asynchronous) | Oral hygiene instructions along with dental aids | Primary: Plaque scores.  Secondary: Oral health beliefs, behaviours,  Gingival bleeding,  DMFT (S), Oral health conditions,  self-efficacy,  adverse effects,  appointment attendance. | Mobile phones are effective in improving adherence to oral hygiene advice in orthodontic patients. | Only orthodontic patients.  Unclear risk of bias |
| Toniazzo et al., 2019*^26^ | 15 (1402)  RCT | Adolescents, adults  Mothers of young children | NR | Orthodontics | Standardized oral hygiene instructions, and educational, informative and motivational messages conveyed by text messages or apps on mobile phones.  (Asynchronous) | Standardized oral hygiene instructions, and patients did not receive text messages | Oral health knowledge, Plaque index, Bleeding index,  Gingival index,  behavioural change | The pooled  SMD for the dental plaque index (n = 10 studies) was −9.43 (95% CI −14.36 to −4.495;  I^2^ = 99%, p < 0.001), and that of gingival bleeding (n = 7 studies) was −8.54 (95% CI  −13.16 to −3.91; I^2^ = 99%, p < 0.001), indicating significant improvement in dental  plaque control and gingival bleeding for groups that received the mobile health  (mHealth) strategy.  mHealth provided significant improvements in reducing dental plaque and gingivitis and may promote better oral health  behaviours. | High variability in follow-up time, risk of bias and parameters to assess outcomes. Studies  included only younger individuals. |
| Torres et al., 2023*^27^ | 4 (277)  Case-control studies | Orthodontic patients | NR | Orthodontics | Remote monitoring.  (Asynchronous) | Intraoral exams, face-to face monitoring, digital models and plaster models | Treatment time, number of refinements, number of refinements aligners, time for the first refinement | With very low certainty of evidence, teledentistry using Dental Monitoring® software is effective as an aid in monitoring the evolution of interceptive orthodontic treatment (high risk of bias) and, especially, treatment performed with aligners (low to moderate risk of bias). The meta-analysis evidenced a reduction in the number of face-to-face appointments (mean difference = −2.75[−3.95, -1.55]; I2 =41%; p=0.04). | The restricted number of studies, the methodological heterogeneity and the very low certainty of the evidence, limit the  generalizability of the results, reducing the possibility  of associating clinical significance regarding the effectiveness of  teledentistry to monitor the evolution of orthodontic treatment in  all the modalities. |
| Troconis et al., 2018^28^ | 4 (757)  NR | Patients in rural areas | Rural settings | Paediatric, dentistry,oral medicine | Intraoral cameras, smartphones and/or webcams.  (Synchronous and asynchronous) | NA | Compliance rates, dental caries screening, treatment completion rates | TD seems to have a positive impact on dental service in rural areas but more evidence is required to considered it as a practical strategy to improve remote consultation between oral health professionals and patients, diagnosis and treatment planning. | Small number of articles, no studies from Latin American or African countries. |
| Uhrin et al, 2023*^29^ | 14 (7913)  Cohort, Cross-sectional and observational studies | Patients with oral lesions, workers of a pipeline factory, patients included from oral screening programs | Rural settings, oral medicine centers, special care clinic | Oral medicine | Photographs sent to specialist via WhatsApp, and a video call, mobile telemedicine application, e-mail.  (Synchronous and asynchronous) | NA | Diagnosis (Sensitivity and specificity, PPV, referral decision) | Using TD tools in the  detection of oral lesions (OLs) showed high specificity (0.92  confidence interval [95% CI] = 0.59–0.99) and sensitivity  (0.93 95% CI = 0.17–1.00). In the differential diagnosis of lesions, there was high sensitivity and specificity (0.942 95% CI = 0.826–0.982 and 0.982 95% CI = 0.913–0.997), respectively.  TD tools could lead to early diagnosis of oral lesions. TD offers a great substitute for face-to-face dental visits in the detection and differential diagnosis of Ols. | Significant heterogeneity among the included articles. Referral decision was not defined in the articles. |
| Wang et al., 2022*^30^ | 12 (2264)  RCT, quasi-experimental study | Parents or caregivers of children aged 12 | Dental clinic | General dentistry | Oral health promotion and be delivered via mobile devices (text messages, mobile apps).  (Asynchronous) | Text messages, mobile apps | Parents’ oral health knowledge, attitude or behaviors and children's oral health-related outcomes | Low/very low certainty of evidence on mHealth approach to improve parents’ oral health knowledge. Lack of evidence on the effects of mHealth intervention exerted on children’s oral health status. | No quantitative analysis of the results due to limited number and heterogeneity of the studies. |

**References:**

1. Abdul NS, Kumari M, Shenoy M, et al. Telemedicine in the diagnosis and management of temporomandibular disorders: a systematic

review conducted according to PRISMA guidelines and the Cochrane Handbook for Systematic Reviews of Interventions. J of Oral

Rehabilitation. Nov 2023;50(11):1340-1347. URL: https://onlinelibrary.wiley.com/toc/ 13652842/50/11 [doi: 10.1111/joor.13546].

2. Alabdullah JH, Daniel SJ. A systematic review on the validity of teledentistry. Telemed J E Health. Aug 2018;24(8):639-648. [doi: 10.1089/tmj.2017.0132] [Medline: 29303678]

3. Al-Moghrabi D, Alkadhimi A, Tsichlaki A, Pandis N, Fleming PS. The influence of mobile applications and social media-based

interventions in producing behavior change among orthodontic patients: a systematic review and meta analysis. Am J Orthod Dentofacial

Orthop. Mar 2022;161(3):338-354. [doi: 10.1016/j.ajodo.2021.09.009] [Medline: 34736817]

4. Aquilanti L, Santarelli A, Mascitti M, Procaccini M, Rappelli G. Dental care access and the elderly: what is the role of teledentistry? A

systematic review. Int J Environ Res Public Health. Dec 4, 2020;17(23):1-13. [doi: 10.3390/ ijerph17239053] [Medline: 33291719]

5. Bhamra IB, Gallagher JE, Patel R. Telehealth technologies in care homes: a gap for dentistry? J Public Health (Oxf). Feb 23,

2024;46(1):e106-e135. [doi: 10.1093/pubmed/fdad258]

6. da Costa CB, Peralta FDS, Ferreira de Mello ALS. How Has Teledentistry Been Applied in Public Dental Health Services? An

Integrative Review. Telemed J E Health 2020;26(7):945-54.

7. Chau RCW, Thu KM, Chaurasia A, Hsung RTC, Lam WYH. A systematic review of the use of mHealth in oral health education among older adults. Dent J (Basel). Aug 8, 2023;11(8):189. [doi: 10.3390/dj11080189] [Medline: 37623285]

8. Choi EM, Park BY, Noh HJ. Efficacy of mobile health care in patients undergoing fixed orthodontic treatment: a systematic review. Int J

Dent Hyg. Feb 2021;19(1):29-38. [doi: 10.1111/idh.12459] [Medline: 32794341]

9. da Silva HEC, Santos GNM, Leite AF, et al. The role of teledentistry in oral cancer patients during the COVID-19 pandemic: an

integrative literature review. Support Care Cancer. Dec 2021;29(12):7209-7223. [doi: 10.1007/s00520 021-06398-0] [Medline: 34219196]

10.De Lima T, Moura ABR, Bezerra PMM, et al. Accuracy of remote examination for detecting potentially malignant oral lesions: a systematic review and meta-analysis. Telemed J E Health. Feb 2024;30(2):381-392. [doi: 10.1089/tmj.2023. 0096] [Medline: 37651222]

11. Emami E, Harnagea H, Shrivastava R, Ahmadi M, Giraudeau N. Patient satisfaction with e-oral health care in rural and remote settings: a systematic review. Syst Rev. 2022;11(1):234. [doi: 10.1186/s13643-022-02103-2]

12. Estai M, Bunt S, Kanagasingam Y, Kruger E, Tennant M. Diagnostic accuracy of teledentistry in the detection of dental caries: a systematic review. J Evid Based Dent Pract. Sep 2016;16(3):161-172. [doi: 10.1016/j.jebdp.2016.08.003] [Medline: 27855831]

13. Estai M, Kanagasingam Y, Tennant M, Bunt S. A systematic review of the research evidence for the benefits of teledentistry. J Telemed Telecare. Apr 2018;24(3):147-156. [doi: 10.1177/1357633X16689433] [Medline: 28118778]

14. Fernández CE, Maturana CA, Coloma SI, Carrasco-Labra A, Giacaman RA. Teledentistry and mHealth for promotion and prevention of oral health: a systematic review and meta-analysis. J Dent Res. Aug 2021;100(9):914-927. [doi: 10. 1177/00220345211003828] [Medline: 33769123]

15. Flores A da C, Lazaro SA, Molina-Bastos CG, et al. Teledentistry in the diagnosis of oral lesions: a systematic review of the literature. J Am Med Inform Assoc. Jul 1, 2020;27(7):1166-1172. [doi: 10.1093/jamia/ocaa069] [Medline: 32568392]

16. Fortich Mesa N, Hoyos Hoyos V. Aplicaciones de la teleodontologia en la practica odontologica revision sistematica/ Applications of teledentistry in dental practice: a systematic review. Rev Fac Odontol Univ Antioq. 2020;32(1). [doi: 10.17533/udea.rfo. v32n1a8]

17. Irving M, Stewart R, Spallek H, Blinkhorn A. Using teledentistry in clinical practice as an enabler to improve access to clinical care: a qualitative systematic review. J Telemed Telecare. Apr 2018;24(3):129-146. [doi: 10.1177/ 1357633X16686776] [Medline: 28092220]

18. Lima IFP, de Andrade Vieira W, de Macedo Bernardino Í, et al. Influence of reminder therapy for controlling bacterial plaque in patients undergoing orthodontic treatment: a systematic review and meta-analysis. Angle Orthod. Jul 2018;88(4):483-493. [doi: 10.2319/111117-770.1] [Medline: 29664334]

19. Lin GSS, Koh SH, Ter KZ, Lim CW, Sultana S, Tan WW. Awareness, knowledge, attitude, and practice of teledentistry among dental practitioners during COVID-19: a systematic review and meta-analysis. Medicina (Kaunas). Jan 15, 2022;58(1):130. [doi: 10.3390/medicina58010130] [Medline: 35056438]

20. Mohammed H, Rizk MZ, Wafaie K, Ulhaq A, Almuzian M. Reminders improve oral hygiene and adherence to appointments in orthodontic patients: a systematic review and meta-analysis. Eur J Orthod. Mar 29, 2019;41(2):204-213. [doi: 10.1093/ejo/cjy045] [Medline: 29947755]

21. Priyank H, Verma A, Zama Khan DU, Prakash Rai N, Kalburgi V, Singh S. Comparative evaluation of dental caries score between teledentistry examination and clinical examination: a systematic review and meta-analysis. Cureus. Jul 2023;15(7):e42414. [doi: 10.7759/cureus.42414] [Medline: 37637546]

22. Rouanet F, Masucci C, Khorn B, Oueiss A, Dridi SM, Charavet C. Pertinence des outils de téléorthodontie : une revue systématique de la littérature. Orthod Fr. Dec 1, 2022;93(4):353-375. [doi: 10.1684/orthodfr.2022.104]

23. Saccomanno S, Quinzi V, Albani A, D’Andrea N, Marzo G, Macchiarelli G. Utility of teleorthodontics in orthodontic emergencies during the COVID-19 pandemic: a systematic review. Healthcare (Basel). Jun 14, 2022;10(6):1108. [doi: 10.3390/healthcare10061108] [Medline: 35742159]

24. Sangalli L, Alessandri-Bonetti A, Dalessandri D. Effectiveness of dental monitoring system in orthodontics: a systematic review. J Orthod. Mar 2024;51(1):28-40. [doi: 10.1177/14653125231178040] [Medline: 37278017]

25. Sharif MO, Newton T, Cunningham SJ. A systematic review to assess interventions delivered by mobile phones in improving adherence to oral hygiene advice for children and adolescents. Br Dent J. Sep 2019;227(5):375-382. [doi: 10. 1038/s41415-019-0660-5] [Medline: 31520040]

26. Toniazzo MP, Nodari D, Muniz F, Weidlich P. Effect of mHealth in improving oral hygiene: a systematic review with meta‐analysis. J Clinic Periodontology. Mar 2019;46(3):297-309. URL: https://onlinelibrary.wiley.com/toc/1600051x/ 46/3 [doi: 10.1111/jcpe.13083]

27. Torres DKB, Santos MCC dos, Normando D. Is teledentistry effective to monitor the evolution of orthodontic treatment? A systematic review and meta-analysis. Dental Press J Orthod. Sep 15, 2023;28(4):e2322195. [doi: 10.1590/2177-6709. 28.4.e2322195.oar]

28. Troconis CM, Ribón JR, et Puello P. Impact of Teledentistry Programs on Dental Service in Rural Areas: A Systematic Review, *International Journal of Applied Engineering Research* (vol. 13, n° 19, pages 14417–1442329.

29. Uhrin E, Domokos Z, Czumbel LM, et al. Teledentistry: a future solution in the diagnosis of oral lesions: diagnostic meta-analysis and systematic review. Telemed J E Health. Nov 2023;29(11):1591-1600. [doi: 10.1089/tmj.2022.0426] [Medline: 36976779]

30. Wang K, Yu KF, Liu P, Lee GHM, Wong MCM. Can mHealth promotion for parents help to improve their children’s oral health? A systematic review. J Dent. Aug 2022;123:104185. [doi: 10.1016/j.jdent.2022.104185] [Medline: 35691452]
