# Supplementary material for: Teledentistry for Improving Access To, and Quality of Oral Health Care: Overview of Systematic Reviews and Meta-Analyses
Source: J Med Internet Res. 2025 Jul 30;27:e65211. doi: 10.2196/65211 (PMC12334114; doi:10.2196/65211)
Supplement: Multimedia Appendix 4 [file jmir-v27-e65211-s004.docx]

**AMSTAR2 results of 30 included studies**

| **Authors, Years** | **Q1** | **Q2** | **Q3** |  | **Q5** | **Q6** | **Q7** | **Q8** | **Q9** | **Q10** | **Q11** | **Q12** | **Q13** | **Q14** | **Q15** | **Q16** | **AMSTAR 2** |
| --- | --- | --- | --- | --- | --- | --- | --- | --- | --- | --- | --- | --- | --- | --- | --- | --- | --- |
| Abdul et al., 2023^1^ | Y | PY | N | PY | N | Y | N | PY | Y | N | No meta | No meta | N | N | No meta | Y | Critically low |
| Alabdullah & Daniel, 2018^2^ | N | PY | N | PY | Y | Y | PY | Y | Y | N | No meta | No meta | N | N | No meta | N | Low |
| Al-Moghrabi et al., 2021*^3^ | Y | Y | N | PY | Y | Y | PY | Y | Y | N | N | Y | N | Y | N | Y | Critically low |
| Aquilanti et al., 2020^4^ | Y | Y | N | PY | Y | Y | PY | Y | N | N | No meta | No meta | N | N | No meta | Y | Critically low |
| Bhamra et al., 2024^5^ | Y | PY | N | PY | N | N | N | PY | Y | N | No meta | No meta | Y | N | No meta | Y | Low |
| BöhmdaCosta et al., 2019^6^ | Y | N | N | PY | Y | Y | PY | PY | N | N | No meta | No meta | N | N | No meta | Y | Critically low |
| Chau et al.,2023^7^ | Y | PY | N | PY | Y | Y | N | PY | Y | N | No meta | No meta | N | N | No meta | Y | Critically low |
| Choi et al., 2021^8^ | Y | PY | N | PY | Y | Y | PY | Y | Y | N | No meta | No meta | Y | N | No meta | Y | Moderate |
| da Silva et al., 2021^9^ | Y | Y | N | PY | Y | Y | PY | Y | Y | N | No meta | No meta | N | N | No meta | Y | Low |
| deLima et al., 2024*^10^ | Y | PY | N | Y | Y | Y | N | Y | Y | N | N | N | N | Y | N | Y | Critically low |
| Emami et al., 2022^11^ | Y | Y | Y | Y | Y | Y | Y | Y | Y | N | No meta | No meta | Y | N | No meta | Y | Moderate |
| **Authors, Years** | **Q1** | **Q2** | **Q3** |  | **Q5** | **Q6** | **Q7** | **Q8** | **Q9** | **Q10** | **Q11** | **Q12** | **Q13** | **Q14** | **Q15** | **Q16** | **AMSTAR 2** |
| Estai et al., 2016^12^ | N | N | N | PY | Y | Y | PY | Y | Y | N | No meta | No meta | Y | N | No meta | Y | Low |
| Estai et al., 2018^13^ | Y | Y | N | PY | Y | Y | PY | Y | N | N | No meta | No meta | Y | N | No meta | Y | Low |
| Fernandez eta l., 2021*^14^ | Y | Y | N | PY | Y | Y | PY | Y | Y | N | Y | Y | Y | Y | N | Y | Low |
| Flores et al., 2020^15^ | Y | Y | N | PY | Y | Y | PY | PY | Y | N | No meta | No meta | N | Y | No meta | Y | Low |
| Fortish-Mesa & Hoyos, 2020^16^ | N | N | N | PY | Y | PY | PY | PY | Y | N | No meta | No meta | N | N | No meta | Y | Critically low |
| Irving et al., 2018^17^ | N | N | N | PY | Y | N | PY | Y | Y | N | No meta | No meta | Y | Y | No meta | Y | Low |
| Lima et al., 2018*^18^ | Y | Y | N | PY | Y | Y | PY | Y | Y | N | Y | Y | N | Y | N | N | Critically low |
| Lin et al., 2022*^19^ | Y | Y | Y | N | Y | Y | PY | Y | Y | N | Y | Y | Y | Y | Y | Y | High |
| Mohammed et al., 2019*^20^ | Y | Y | N | Y | Y | Y | PY | Y | Y | N | Y | Y | Y | Y | N | Y | Low |
| Priyank et al., 2023*^21^ | Y | PY | N | PY | N | N | N | N | Y | N | N | N | Y | Y | N | Y | Critically low |
| Rouanet et al. 2022^22^ | Y | PY | N | N | Y | Y | N | PY | Y | N | No meta | No meta | N | N | No meta | Y | Critically low |
| Saccomanno et al., 2022^23^ | Y | Y | N | PY | Y | Y | N | PY | N | N | No meta | No meta | N | N | No meta | Y | Critically low |
| Sangalli et al., 2023^24^ | Y | Y | Y | PY | Y | N | Y | Y | Y | N | Nometa | Nometa | Y | N | No meta | Y | Moderate |
| **Authors, Years** | **Q1** | **Q2** | **Q3** |  | **Q5** | **Q6** | **Q7** | **Q8** | **Q9** | **Q10** | **Q11** | **Q12** | **Q13** | **Q14** | **Q15** | **Q16** | **AMSTAR 2** |
| Sharif et al., 2019^25^ | Y | Y | N | Y | Y | N | PY | Y | Y | Y | No meta | No meta | Y | Y | No meta | Y | Moderate |
| Toniazzo et al., 2019*^26^ | Y | N | N | PY | Y | Y | PY | Y | Y | N | Y | Y | N | Y | N | Y | Critically low |
| Torres et al., 2023*^27^ | Y | N | Y | PY | Y | Y | PY | PY | Y | N | N | Y | Y | Y | N | Y | Critically low |
| Troconis et al., 2018^28^ | Y | N | N | PY | N | N | N | PY | Y | N | No meta | No meta | N | N | No meta | N | Critically low |
| Uhrin et al, 2023*^29^ | Y | Y | N | PY | Y | Y | Y | PY | Y | N | Y | N | N | Y | N | Y | Critically low |
| Wang et al., 2022*^30^ | Y | Y | N | PY | Y | Y | PY | Y | Y | N | Y | Y | Y | Y | N | Y | Low |

Legend:

Y: Yes N: No PY: Partial yes No meta: No meta-analysis

**References**

1. Abdul NS, Kumari M, Shenoy M, et al. Telemedicine in the diagnosis and management of temporomandibular disorders: a systematic

review conducted according to PRISMA guidelines and the Cochrane Handbook for Systematic Reviews of Interventions. J of Oral

Rehabilitation. Nov 2023;50(11):1340-1347. URL: https://onlinelibrary.wiley.com/toc/ 13652842/50/11 [doi: 10.1111/joor.13546].

2. Alabdullah JH, Daniel SJ. A systematic review on the validity of teledentistry. Telemed J E Health. Aug 2018;24(8):639-648. [doi: 10.1089/tmj.2017.0132] [Medline: 29303678]

3. Al-Moghrabi D, Alkadhimi A, Tsichlaki A, Pandis N, Fleming PS. The influence of mobile applications and social media-based

interventions in producing behavior change among orthodontic patients: a systematic review and meta analysis. Am J Orthod Dentofacial

Orthop. Mar 2022;161(3):338-354. [doi: 10.1016/j.ajodo.2021.09.009] [Medline: 34736817]

4. Aquilanti L, Santarelli A, Mascitti M, Procaccini M, Rappelli G. Dental care access and the elderly: what is the role of teledentistry? A

systematic review. Int J Environ Res Public Health. Dec 4, 2020;17(23):1-13. [doi: 10.3390/ ijerph17239053] [Medline: 33291719]

5. Bhamra IB, Gallagher JE, Patel R. Telehealth technologies in care homes: a gap for dentistry? J Public Health (Oxf). Feb 23,

2024;46(1):e106-e135. [doi: 10.1093/pubmed/fdad258]

6. da Costa CB, Peralta FDS, Ferreira de Mello ALS. How Has Teledentistry Been Applied in Public Dental Health Services? An

Integrative Review. Telemed J E Health 2020;26(7):945-54.

7. Chau RCW, Thu KM, Chaurasia A, Hsung RTC, Lam WYH. A systematic review of the use of mHealth in oral health education among older adults. Dent J (Basel). Aug 8, 2023;11(8):189. [doi: 10.3390/dj11080189] [Medline: 37623285]

8. Choi EM, Park BY, Noh HJ. Efficacy of mobile health care in patients undergoing fixed orthodontic treatment: a systematic review. Int J

Dent Hyg. Feb 2021;19(1):29-38. [doi: 10.1111/idh.12459] [Medline: 32794341]

9. da Silva HEC, Santos GNM, Leite AF, et al. The role of teledentistry in oral cancer patients during the COVID-19 pandemic: an

integrative literature review. Support Care Cancer. Dec 2021;29(12):7209-7223. [doi: 10.1007/s00520 021-06398-0] [Medline: 34219196]

10. de Lima T, Moura ABR, Bezerra PMM, et al. Accuracy of remote examination for detecting potentially malignant oral lesions: a systematic review and meta-analysis. Telemed J E Health. Feb 2024;30(2):381-392. [doi: 10.1089/tmj.2023. 0096] [Medline: 37651222]

11. Emami E, Harnagea H, Shrivastava R, Ahmadi M, Giraudeau N. Patient satisfaction with e-oral health care in rural and remote settings: a systematic review. Syst Rev. 2022;11(1):234. [doi: 10.1186/s13643-022-02103-2]

12. Estai M, Bunt S, Kanagasingam Y, Kruger E, Tennant M. Diagnostic accuracy of teledentistry in the detection of dental caries: a systematic review. J Evid Based Dent Pract. Sep 2016;16(3):161-172. [doi: 10.1016/j.jebdp.2016.08.003] [Medline: 27855831]

13. Estai M, Kanagasingam Y, Tennant M, Bunt S. A systematic review of the research evidence for the benefits of teledentistry. J Telemed Telecare. Apr 2018;24(3):147-156. [doi: 10.1177/1357633X16689433] [Medline: 28118778]

14. Fernández CE, Maturana CA, Coloma SI, Carrasco-Labra A, Giacaman RA. Teledentistry and mHealth for promotion and prevention of oral health: a systematic review and meta-analysis. J Dent Res. Aug 2021;100(9):914-927. [doi: 10. 1177/00220345211003828] [Medline: 33769123]

15. Flores A da C, Lazaro SA, Molina-Bastos CG, et al. Teledentistry in the diagnosis of oral lesions: a systematic review of the literature. J Am Med Inform Assoc. Jul 1, 2020;27(7):1166-1172. [doi: 10.1093/jamia/ocaa069] [Medline: 32568392]

16. Fortich Mesa N, Hoyos Hoyos V. Aplicaciones de la teleodontologia en la practica odontologica revision sistematica/ Applications of teledentistry in dental practice: a systematic review. Rev Fac Odontol Univ Antioq. 2020;32(1). [doi: 10.17533/udea.rfo. v32n1a8]

17. Irving M, Stewart R, Spallek H, Blinkhorn A. Using teledentistry in clinical practice as an enabler to improve access to clinical care: a qualitative systematic review. J Telemed Telecare. Apr 2018;24(3):129-146. [doi: 10.1177/ 1357633X16686776] [Medline: 28092220]

18. Lima IFP, de Andrade Vieira W, de Macedo Bernardino Í, et al. Influence of reminder therapy for controlling bacterial plaque in patients undergoing orthodontic treatment: a systematic review and meta-analysis. Angle Orthod. Jul 2018;88(4):483-493. [doi: 10.2319/111117-770.1] [Medline: 29664334]

19. Lin GSS, Koh SH, Ter KZ, Lim CW, Sultana S, Tan WW. Awareness, knowledge, attitude, and practice of teledentistry among dental practitioners during COVID-19: a systematic review and meta-analysis. Medicina (Kaunas). Jan 15, 2022;58(1):130. [doi: 10.3390/medicina58010130] [Medline: 35056438]

20. Mohammed H, Rizk MZ, Wafaie K, Ulhaq A, Almuzian M. Reminders improve oral hygiene and adherence to appointments in orthodontic patients: a systematic review and meta-analysis. Eur J Orthod. Mar 29, 2019;41(2):204-213. [doi: 10.1093/ejo/cjy045] [Medline: 29947755]

21. Priyank H, Verma A, Zama Khan DU, Prakash Rai N, Kalburgi V, Singh S. Comparative evaluation of dental caries score between teledentistry examination and clinical examination: a systematic review and meta-analysis. Cureus. Jul 2023;15(7):e42414. [doi: 10.7759/cureus.42414] [Medline: 37637546]

22. Rouanet F, Masucci C, Khorn B, Oueiss A, Dridi SM, Charavet C. Pertinence des outils de téléorthodontie : une revue systématique de la littérature. Orthod Fr. Dec 1, 2022;93(4):353-375. [doi: 10.1684/orthodfr.2022.104]

23. Saccomanno S, Quinzi V, Albani A, D’Andrea N, Marzo G, Macchiarelli G. Utility of teleorthodontics in orthodontic emergencies during the COVID-19 pandemic: a systematic review. Healthcare (Basel). Jun 14, 2022;10(6):1108. [doi: 10.3390/healthcare10061108] [Medline: 35742159]

24. Sangalli L, Alessandri-Bonetti A, Dalessandri D. Effectiveness of dental monitoring system in orthodontics: a systematic review. J Orthod. Mar 2024;51(1):28-40. [doi: 10.1177/14653125231178040] [Medline: 37278017]

25. Sharif MO, Newton T, Cunningham SJ. A systematic review to assess interventions delivered by mobile phones in improving adherence to oral hygiene advice for children and adolescents. Br Dent J. Sep 2019;227(5):375-382. [doi: 10. 1038/s41415-019-0660-5] [Medline: 31520040]

26. Toniazzo MP, Nodari D, Muniz F, Weidlich P. Effect of mHealth in improving oral hygiene: a systematic review with meta‐analysis. J Clinic Periodontology. Mar 2019;46(3):297-309. URL: https://onlinelibrary.wiley.com/toc/1600051x/ 46/3 [doi: 10.1111/jcpe.13083]

27. Torres DKB, Santos MCC dos, Normando D. Is teledentistry effective to monitor the evolution of orthodontic treatment? A systematic review and meta-analysis. Dental Press J Orthod. Sep 15, 2023;28(4):e2322195. [doi: 10.1590/2177-6709. 28.4.e2322195.oar]

28. Troconis CM, Ribón JR, et Puello P. Impact of Teledentistry Programs on Dental Service in Rural Areas: A Systematic Review,  *International Journal of Applied Engineering Research* (vol. 13, n° 19, pages 14417–1442329.

29. Uhrin E, Domokos Z, Czumbel LM, et al. Teledentistry: a future solution in the diagnosis of oral lesions: diagnostic meta-analysis and systematic review. Telemed J E Health. Nov 2023;29(11):1591-1600. [doi: 10.1089/tmj.2022.0426] [Medline: 36976779]

30. Wang K, Yu KF, Liu P, Lee GHM, Wong MCM. Can mHealth promotion for parents help to improve their children’s oral health? A systematic review. J Dent. Aug 2022;123:104185. [doi: 10.1016/j.jdent.2022.104185] [Medline: 35691452]
