# Supplementary material for: Teledentistry for Improving Access To, and Quality of Oral Health Care: Overview of Systematic Reviews and Meta-Analyses
Source: J Med Internet Res. 2025 Jul 30;27:e65211. doi: 10.2196/65211 (PMC12334114; doi:10.2196/65211)
Supplement: Multimedia Appendix 7 [file jmir-v27-e65211-s007.docx]

**Overlap for SRs with meta-analysis**


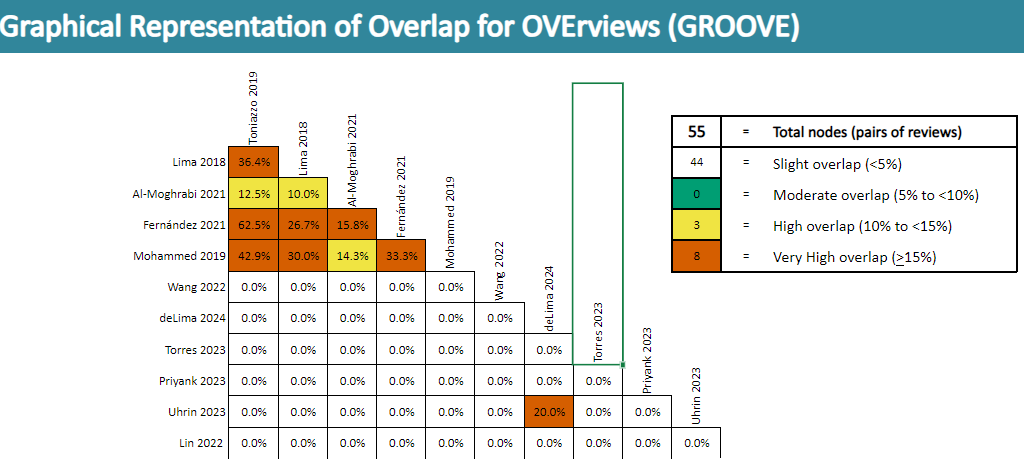


| Number of columns (number of reviews) | c | 11 |
| --- | --- | --- |
| Number of rows (number of index publications) | r | 42 |
| Number of included primary studies (including double counting) | N | 66 |
| Covered area | N/(rc) | 14.29% |
| Corrected covered area | (N-r)/(rc-r) | 5.71% |
| Interpretation of overlap | **Moderate overlap** | |
| Structural Zeros | X | 0 |
| Corrected covered area  (adjusting by structural zeros) | (N-r)/(rc-r-X) | 5.71% |

**References**

1. Al-Moghrabi D, Alkadhimi A, Tsichlaki A, Pandis N, Fleming PS. The influence of mobile applications and social media-based interventions in producing behavior change among orthodontic patients: a systematic review and meta analysis. Am J Orthod Dentofacial Orthop. Mar 2022;161(3):338-354. [doi: 10.1016/j.ajodo.2021.09.009] [Medline: 34736817]

2. de Lima T, Moura ABR, Bezerra PMM, et al. Accuracy of remote examination for detecting potentially malignant oral lesions: a systematic review and meta-analysis. Telemed J E Health. Feb 2024;30(2):381-392. [doi: 10.1089/tmj.2023. 0096] [Medline: 37651222]

3. Fernández CE, Maturana CA, Coloma SI, Carrasco-Labra A, Giacaman RA. Teledentistry and mHealth for promotion and prevention of oral health: a systematic review and meta-analysis. J Dent Res. Aug 2021;100(9):914-927. [doi: 10. 1177/00220345211003828] [Medline: 33769123]

4.Lima IFP, de Andrade Vieira W, de Macedo Bernardino Í, et al. Influence of reminder therapy for controlling bacterial plaque in patients undergoing orthodontic treatment: a systematic review and meta-analysis. Angle Orthod. Jul 2018;88(4):483-493. [doi: 10.2319/111117-770.1] [Medline: 29664334]

5. Lin GSS, Koh SH, Ter KZ, Lim CW, Sultana S, Tan WW. Awareness, knowledge, attitude, and practice of teledentistry among dental practitioners during COVID-19: a systematic review and meta-analysis. Medicina (Kaunas). Jan 15, 2022;58(1):130. [doi: 10.3390/medicina58010130] [Medline: 35056438]

6. Mohammed H, Rizk MZ, Wafaie K, Ulhaq A, Almuzian M. Reminders improve oral hygiene and adherence to appointments in orthodontic patients: a systematic review and meta-analysis. Eur J Orthod. Mar 29, 2019;41(2):204-213. [doi: 10.1093/ejo/cjy045] [Medline: 29947755]

7. Priyank H, Verma A, Zama Khan DU, Prakash Rai N, Kalburgi V, Singh S. Comparative evaluation of dental caries score between teledentistry examination and clinical examination: a systematic review and meta-analysis. Cureus. Jul 2023;15(7):e42414. [doi: 10.7759/cureus.42414] [Medline: 37637546]

8. Toniazzo MP, Nodari D, Muniz F, Weidlich P. Effect of mHealth in improving oral hygiene: A systematic review with meta-analysis. J Clin Periodontol 2019;46(3):297-309.

9. Torres DKB, Santos MCC dos, Normando D. Is teledentistry effective to monitor the evolution of orthodontic treatment? A systematic review and meta-analysis. Dental Press J Orthod. Sep 15, 2023;28(4):e2322195. [doi: 10.1590/2177-6709. 28.4.e2322195.oar]

10. Uhrin E, Domokos Z, Czumbel LM, et al. Teledentistry: a future solution in the diagnosis of oral lesions: diagnostic meta-analysis and systematic review. Telemed J E Health. Nov 2023;29(11):1591-1600. [doi: 10.1089/tmj.2022.0426] [Medline: 36976779]

11. Wang K, Yu KF, Liu P, Lee GHM, Wong MCM. Can mHealth promotion for parents help to improve their children’s oral health? A systematic review. J Dent. Aug 2022;123:104185. [doi: 10.1016/j.jdent.2022.104185] [Medline: 35691452]
