# Supplementary material for: Teledentistry for Improving Access To, and Quality of Oral Health Care: Overview of Systematic Reviews and Meta-Analyses
Source: J Med Internet Res. 2025 Jul 30;27:e65211. doi: 10.2196/65211 (PMC12334114; doi:10.2196/65211)
Supplement: Multimedia Appendix 9 [file jmir-v27-e65211-s009.docx]

**Overlap for SRs including plaque index outcomes**

**
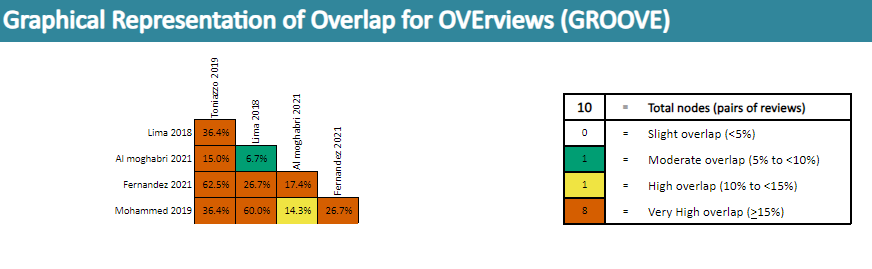
**

| Number of columns (number of reviews) | c | 5 |
| --- | --- | --- |
| Number of rows (number of index publications) | r | 24 |
| Number of included primary studies (including double counting) | N | 46 |
| Covered area | N/(rc) | 38.33% |
| Corrected covered area | (N-r)/(rc-r) | 22.92% |
| Interpretation of overlap | **Very High overlap** | |
| Structural Zeros | X | 0 |
| Corrected covered area  (adjusting by structural zeros) | (N-r)/(rc-r-X) | 22.92% |

**References**

1. Al-Moghrabi D, Alkadhimi A, Tsichlaki A, Pandis N, Fleming PS. The influence of mobile applications and social media-based interventions in producing behavior change among orthodontic patients: a systematic review and meta analysis. Am J Orthod Dentofacial Orthop. Mar 2022;161(3):338-354. [doi: 10.1016/j.ajodo.2021.09.009] [Medline: 34736817]

2. Fernández CE, Maturana CA, Coloma SI, Carrasco-Labra A, Giacaman RA. Teledentistry and mHealth for promotion and prevention of oral health: a systematic review and meta-analysis. J Dent Res. Aug 2021;100(9):914-927. [doi: 10. 1177/00220345211003828] [Medline: 33769123]

3.Lima IFP, de Andrade Vieira W, de Macedo Bernardino Í, et al. Influence of reminder therapy for controlling bacterial plaque in patients undergoing orthodontic treatment: a systematic review and meta-analysis. Angle Orthod. Jul 2018;88(4):483-493. [doi: 10.2319/111117-770.1] [Medline: 29664334]

4.Mohammed H, Rizk MZ, Wafaie K, Ulhaq A, Almuzian M. Reminders improve oral hygiene and adherence to appointments in orthodontic patients: a systematic review and meta-analysis. Eur J Orthod. Mar 29, 2019;41(2):204-213. [doi: 10.1093/ejo/cjy045] [Medline: 29947755]

5. 5. Toniazzo MP, Nodari D, Muniz F, Weidlich P. Effect of mHealth in improving oral hygiene: a systematic review with meta‐analysis. J Clinic Periodontology. Mar 2019;46(3):297-309. URL: https://onlinelibrary.wiley.com/toc/1600051x/ 46/3 [doi: 10.1111/jcpe.13083
