# Supplementary material for: Teledentistry for Improving Access To, and Quality of Oral Health Care: Overview of Systematic Reviews and Meta-Analyses
Source: J Med Internet Res. 2025 Jul 30;27:e65211. doi: 10.2196/65211 (PMC12334114; doi:10.2196/65211)
Supplement: Multimedia Appendix 10 [file jmir-v27-e65211-s010.docx]

**Overlap for SRs including White spots outcomes**


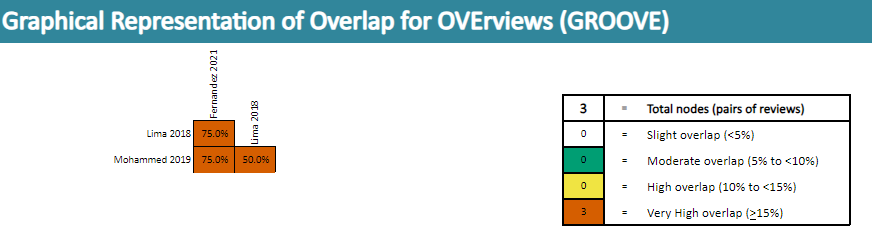


| Number of columns (number of reviews) | c | 3 |
| --- | --- | --- |
| Number of rows (number of index publications) | r | 4 |
| Number of included primary studies (including double counting) | N | 10 |
| Covered area | N/(rc) | 83.33% |
| Corrected covered area | (N-r)/(rc-r) | 75.00% |
| Interpretation of overlap | **Very High overlap** | |
| Structural Zeros | X | 0 |
| Corrected covered area  (adjusting by structural zeros) | (N-r)/(rc-r-X) | 75.00% |

**References**

1. Fernández CE, Maturana CA, Coloma SI, Carrasco-Labra A, Giacaman RA. Teledentistry and mHealth for promotion and prevention of oral health: a systematic review and meta-analysis. J Dent Res. Aug 2021;100(9):914-927. [doi: 10. 1177/00220345211003828] [Medline: 33769123]

2. Lima IFP, de Andrade Vieira W, de Macedo Bernardino Í, et al. Influence of reminder therapy for controlling bacterial plaque in patients undergoing orthodontic treatment: a systematic review and meta-analysis. Angle Orthod. Jul 2018;88(4):483-493. [doi: 10.2319/111117-770.1] [Medline: 29664334]

3. Mohammed H, Rizk MZ, Wafaie K, Ulhaq A, Almuzian M. Reminders improve oral hygiene and adherence to appointments in orthodontic patients: a systematic review and meta-analysis. Eur J Orthod. Mar 29, 2019;41(2):204-213. [doi: 10.1093/ejo/cjy045] [Medline: 29947755]
